# Describing Directional Cell Migration with a Characteristic Directionality Time

**DOI:** 10.1371/journal.pone.0127425

**Published:** 2015-05-20

**Authors:** Alex J. Loosley, Xian M. O’Brien, Jonathan S. Reichner, Jay X. Tang

**Affiliations:** 1 Department of Physics, Brown University, Providence, RI, USA; 2 Division of Surgical Research, Department of Surgery, Rhode Island Hospital and the Warren Alpert Medical School of Brown University, Providence, RI, USA; King’s College London, UNITED KINGDOM

## Abstract

Many cell types can bias their direction of locomotion by coupling to external cues. Characteristics such as how fast a cell migrates and the directedness of its migration path can be quantified to provide metrics that determine which biochemical and biomechanical factors affect directional cell migration, and by how much. To be useful, these metrics must be reproducible from one experimental setting to another. However, most are not reproducible because their numerical values depend on technical parameters like sampling interval and measurement error. To address the need for a reproducible metric, we analytically derive a metric called directionality time, the minimum observation time required to identify motion as directionally biased. We show that the corresponding fit function is applicable to a variety of ergodic, directionally biased motions. A motion is ergodic when the underlying dynamical properties such as speed or directional bias do not change over time. Measuring the directionality of nonergodic motion is less straightforward but we also show how this class of motion can be analyzed. Simulations are used to show the robustness of directionality time measurements and its decoupling from measurement errors. As a practical example, we demonstrate the measurement of directionality time, step-by-step, on noisy, nonergodic trajectories of chemotactic neutrophils. Because of its inherent generality, directionality time ought to be useful for characterizing a broad range of motions including intracellular transport, cell motility, and animal migration.

## Introduction

Directional cell migration is the process in which a single cell or a group of cells bias their direction of locomotion by coupling to an external cue. External cues may be soluble in nature such as during chemotaxis [1], insoluble such as during haptotaxis [2], or mechanical such as during durotaxis [3] and gravitaxis [4]. Processes involving directional cell migration are ubiquitous in nature and essential for many fundamental biological processes facilitating the innate and adaptive immune systems [5, 6], sexual reproduction [7], embryonic development [8], cancer metastasis [9, 10], and more. The efficacy to which cells are able to carry out these functions is often tied to the characteristics of their migration, including migration speed, persistence, and tortuosity [11]. These characteristics can be quantified to determine which biochemical and biomechanical factors affect cell migration, and by how much, but choosing the right metric is important. To introduce this work, we briefly review several commonly used metrics for characterizing cell migration to show that the current paradigm is good for characterizing persistent motion in the absence of an external cue, but does not reproducibly characterize directional motion. The main goal of this work is to derive an intuitive metric for reproducibly quantifying the directional bias of motion, independent of persistence.

### Commonly Used Analytical Tools For Characterizing Migration

Mean squared displacement (MSD) is a common metric for measuring migration speed and distance traveled because it is easily interpretable and readily derived from mathematical models of motion. Numerous studies that characterize directional migration use MSD in conjunction with at least one other metric for quantifying path persistence or tortuosity [11–17]. Three examples of such metrics used are: the distribution of turning angles between discrete measurements of centroid displacements (turning angle distribution, TAD); tortuosity (also known as straightness index [18, 19], chemotactic index [15, 20], or directionality ratio [21]) defined as the end-to-end distance traveled divided by the total migration path length; and tangent-tangent correlation, which describes the correlation in migration path orientation over a specified length or time interval.

### Sampling Interval Dependence

In order to gain insight from quantitative characterizations of the migration path, the numerical values of the metrics applied must be reproducible from one set of experiments to another. Such values should also reflect the true kinematic properties of migration by decoupling from pseudo random kinematics induced by measurement error along the migration path. One common shortcoming of TAD, tortuosity, and tangent-tangent correlation is that they each implicitly depend on sampling interval, Δ*t*, which is the time interval between position measurements. Sampling interval can be chosen arbitrarily, implying that TAD, tortuosity, and tangent-tangent correlation curves are not generally reproducible without using an equivalent sampling interval across all experiments. Even when sampling intervals are accounted for, a sampling interval dependent metric only characterizes migration at an arbitrarily chosen time scale at which the observable may or may not decouple from measurement error.

**Fig 1:**
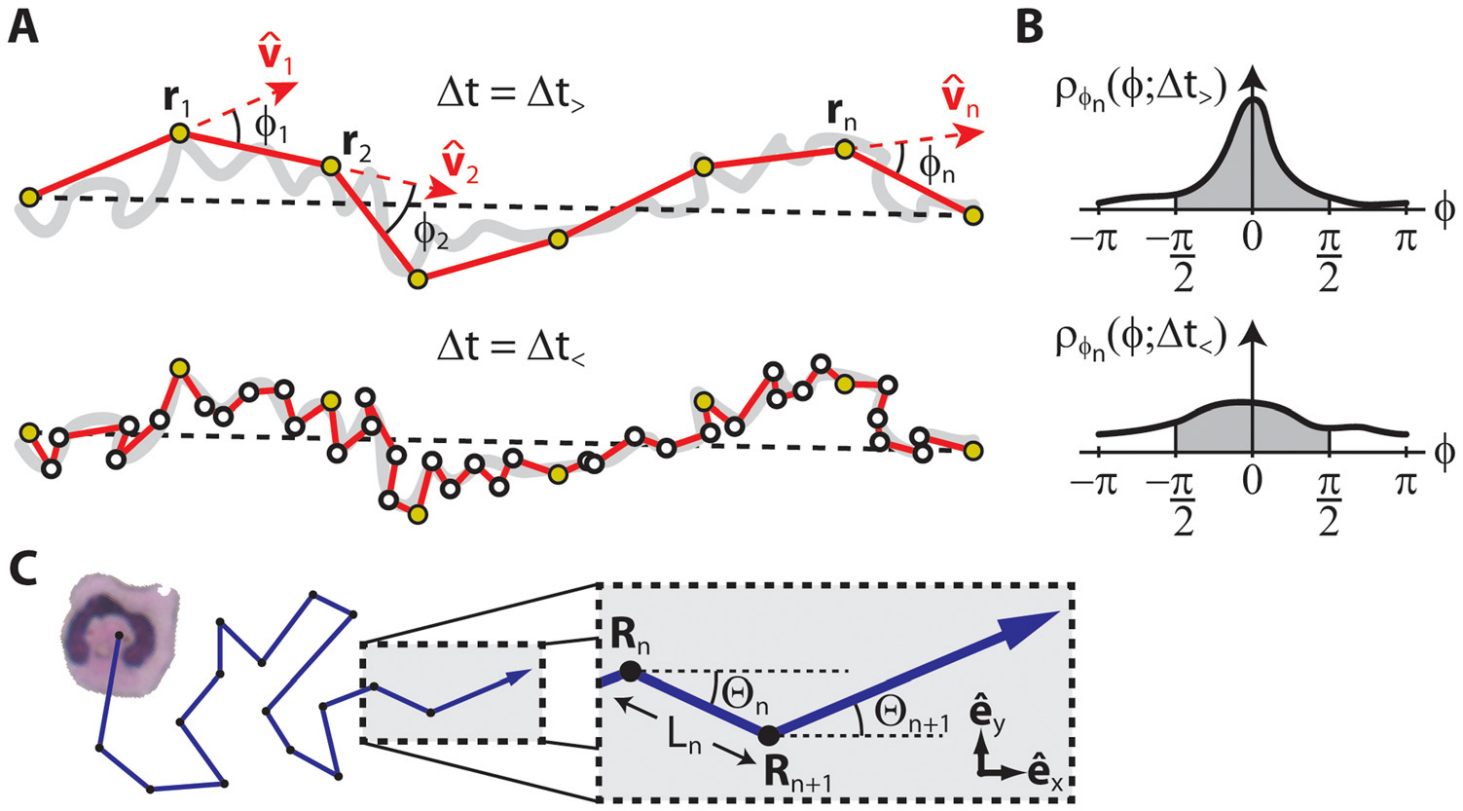
The effects of sampling interval on measurements and characterization of migration trajectories. (A) A migration path sampled with a long sampling interval, Δ*t* = Δ*t*_>_ (top, outlined yellow circles), and a short sampling interval, Δ*t* = Δ*t*_<_ (bottom, outlined white circles). Using the long sampling interval diagram, the observed migration path is formed by connecting measurements of centroid positions, **r**_1_, **r**_2_, …, with lines (in red). Unit tangent vectors are shown as ${\hat{\text{v}}}_{1},{\hat{\text{v}}}_{2},...$ while turning angles, *ϕ*_1_, *ϕ*_2_, …, are defined as the angle between successive tangent vectors. Differences in centroid position from the true migration path (thick grey curve) represent measurement error. (B) Turning angle distributions (TAD), *ρ*_*ϕ*_*n*__(*ϕ*;Δ*t*), based on both the long (top) and short (bottom) sampling intervals. A measure of migration persistence known as TAD persistence is the area under the TAD curve between $\pm \frac{\pi }{2}$ (shaded). TAD persistence depends notably on sampling interval. Similar diagrams can be used to show the sampling interval dependence of metrics such as tortuosity and tangent-tangent correlation. (C) A diagram visually defining terms necessary to analytically derive the directionality time model. A cell is depicted as a random walker with steps (**R**_1_, **R**_2_, …, **R**_*n*_, …) located in a 2-D coordinate system defined by the unit vectors ${\hat{\text{e}}}_{x}$ and ${\hat{\text{e}}}_{y}$. Capital letters correspond to random variables. For each step, there is a corresponding step length *L*_*n*_ and polar angle Θ_*n*_. The latter is defined with respect to unit vector ${\hat{\text{e}}}_{x}$ and should not be confused with the corresponding turning angle, Φ_*n*_ = Θ_*n*_ − Θ_*n*−1_.

To visualize sampling interval dependence, consider two experimental measurements of a migration path, one using a “long” sampling interval, Δ*t* = Δ*t*_>_, and the other using a “short” sampling interval, Δ*t* = Δ*t*_<_ (Fig 1A, top and bottom, respectively). Circles are centroid positions and the corresponding perceived migration paths (red line segments connecting circles) are juxtaposed against the true migration path (thick grey curve). The deviation between centroid positions (**r**_*n*_ = **r**(*t*_*n*_), where *t*_*n*_ = *n*Δ*t*, *n* = 1, 2, …, *N*, Δ*t* = Δ*t*_<_ or Δ*t*_>_) and the true migration path represents centroid measurement error, which depends on factors such as image resolution and cell boundary detection accuracy. Angles between successive red line segments, *ϕ*_*n*_, are turning angles (−*π* < *ϕ* ≤ *π*). Taking into account all turning angles, the normalized TAD, *ρ*_*ϕ*_*n*__(*ϕ*; Δ*t*), is calculated for both sampling intervals (Fig 1B). As the sampling interval increases towards the total duration of the migration path, the TAD curve becomes sharply peaked at *ϕ* = 0. Conversely, as the sampling interval decreases towards zero, the effects of diffusive motion and centroid measurement error flatten the TAD curve. Hence, TAD depends notably on sampling interval. One measure of persistence is the so-called turning angle persistence (TAD persistence), the fraction of all turning angles between $\pm \frac{\pi }{2}$ (shaded area under TAD curves in Fig 1B). TAD persistence depends on the sampling interval just as TAD does.

The dependence of tortuosity on sampling interval is apparent when considering the limit that the sampling interval approaches the total duration of the migration path. In this limit, total path length is the end-to-end length resulting in a tortuosity of 1. When the sampling interval decreases, the total path length increases due to centroid measurement error and the underlying fractal nature of the migration path itself [19, 22, 23].

### Sampling Interval Independent Metrics

Tangent-tangent correlation, $\bar{\hat{v}(t+\tau )\cdot \hat{v}(t)}$, is the time averaged cosine of the angle between tangent vectors $\hat{\text{v}}(t+\tau )$ and $\hat{\text{v}}(t)$ that are separated by a time interval *τ*. The overline denotes an average over all time *t*. When applied to discrete experimental data captured at a specific sampling interval, $\hat{\text{v}}(t)$ is replaced by ${\hat{\text{v}}}_{n}$ (Fig 1A). Tangent-tangent curves are sampling interval dependent because the difference between the true instantaneous tangent vector and the measured tangent vector generally increases as the sampling interval increases. However, there is a sampling interval independent measure called persistence time that derives from the tangent-tangent correlation curve. Persistence time, *t*_*p*_, is the time scale below which directional orientation of the migration path remains correlated. In general, persistence time is measured as a fit parameter in a model that fits the tangent-tangent correlation curve over all time scales. Therefore, measurements of persistence time are sampling interval independent so long as the sampling interval is small enough to provide enough data points for a good fit.

Persistence time and migration speed together are sufficient to characterize non-directional motion but an additional metric is needed when motion is directional (biased by an external cue). This third metric should be sampling interval independent, like persistence time, and decouple from measurement error. Such a metric is derived in this article.

Sampling interval independence implies an integration of data over all time intervals rather than choosing to make a measurement based on one specific sampling interval. One approach to achieving a sampling interval independent metric of directionality could entail fitting data such as TAD persistence or tortuosity to a model, thereby measuring a set of fit parameters. However, TAD persistence and tortuosity models are difficult to calculate and interpret. MSD models are easier to calculate and interpret and several models have already been analytically derived [19, 24, 25]. Nevertheless, this approach of fitting to a model only works if the underlying kinematics of the migration are understood *a priori* such that a model can be chosen. While one can attempt to fit more than one model to determine which fits best, changes to MSD from one model to the next can be small with respect to the error bars on an experimental MSD curve. Hence, there is the possibility of a causality loop—one cannot accurately understand a set of migration paths without knowledge of the underlying process and corresponding random walk model, but one is unsure of the corresponding random walk model without understanding the migration paths.

To circumvent this causality loop, we take a bottom up approach to derive a generalized sampling interval independent metric called directionality time. Whereas persistence time is the time scale below which the orientation of the migration path remains correlated, directionality time is defined as the time scale above which the orientation of the migration path becomes correlated due to an external directional bias. Directionality time supplements persistence time when characterizing directional random walk-like motion. We derive a directionality time fit model that is broadly applicable to many types of ergodic directional motion and decouples from measurement error. We also discuss how the directionality time fit model can be adapted to handle heterogeneous populations of random walkers and nonergodic motion. Finally, we demonstrate its implementation on data of chemotactic neutrophils that migrate directionally with non uniform speed.

## Methods

This section contains three subsections reviewing ensemble averaged squared displacement, mean squared displacement (MSD), and ergodicity, followed by two subsections with general methods information. The concepts reviewed here are necessary to conceptually derive directionality time. The models contained within this article are derived in full mathematical detail in the supporting information (Appendices A and B in S1 File).

### Mean Squared Displacement

We begin with the assertion that ensemble averaged squared displacement follows a power law

$$\begin{array}{c} \langle r_{i}^{2}\left(t\right)\rangle ∼t^{\alpha } \end{array}$$ (1)

where *i* = 1, 2, 3, … is the index of one migration path in an ensemble, and the angle brackets ⟨⟩ denote the ensemble average over squared displacements measured at time *t*. To be precise about the type of averaging, we call this quantity the ensemble averaged squared displacement (EASD) instead of MSD. The exponent *α* characterizes the motion. A constant value of *α* = 1 indicates diffusive (random) motion whereas *α* = 2 indicates ballistic (directional) motion. Other values represent subdiffusive motion (0 < *α* < 1), superdiffusive motion (1 < *α* < 2), or no motion at all (*α* = 0).

When few sets of trajectories are available for the ensemble average, time averaged squared displacement (TASD) can be calculated to reduce statistical noise. The TASD of the *i* ^th^ migration path is given by

$$\begin{array}{c} \bar{r_{i}^{2}}\left(\tau \right)=\bar{{\left|r_{i}\left(t+\tau \right)-r_{i}\left(t\right)\right|}^{2}} \end{array}$$ (2)

where the overline denotes an average over time *t*, leaving TASD a function of the time interval, *τ*. Squared displacement that is first time averaged and then ensemble averaged, $\left\langle \bar{r^{2}}(\tau )\right\rangle$, is hereby referred to as MSD as this is how it is often defined in other studies [14, 17, 26].

### Ergodicity

Ergodicity, *ξ*_*i*_, is the proportionality factor between time averaged squared displacement of the *i* ^th^ migration path (TASD) and ensemble averaged squared displacement (EASD):

$$\begin{array}{c} \bar{r_{i}^{2}}=\xi_{i}\left\langle r^{2}\right\rangle \end{array}$$ (3)

A motion is ergodic when the underlying dynamical properties such as cell speed or directional bias do not change over time. Mathematically, this corresponds to *ξ* = 1 over all time, indicating no difference between the TASD and EASD. This equivalence is useful because TASD measurements can be used to represent EASD measurements when the latter is statistically noisy. In general, time averaging smooths out complexities in the squared displacement caused by variables that change over time (and space), such as instantaneous speed. When such factors change significantly as is often the case with collective and cooperative motion, and motion through confined, spatially heterogeneous topologies (*e.g*. nonuniform substrates where environmental factors such as adhesiveness vary over time and space), ⟨*ξ*⟩ ≠ 1 and the migration paths are said to be nonergodic. Nonergodic motion is mathematically more difficult to characterize and this has implications on directionality time measurements.

### Characterizing Motion using the Slope of Mean Squared Displacement in log-log Coordinates

The slope of EASD plotted in log-log coordinates is an approximate measure of the EASD exponent *α* (Eq 1) and therefore a measure that characterizes trajectory diffusivity and/or directedness. Using Eq 1, and noting that *α* can change with time *t*, one can define the log-log EASD slope: $\beta (t)=\frac{\text{d}\alpha }{\text{d}t}t\ln t+\alpha$. When EASD exponent *α* is constant, *β* = *α*. Otherwise, *β*(*t*) is an estimator of *α*(*t*), and therefore an estimator of how diffusive or ballistic the motion is at a particular time, *t*. In the case where the migration paths are ergodic, EASD and MSD are interchangeable and the log-log MSD slope *β*(*τ*) can also be calculated to characterize motion as a function of time interval *τ*. In this work, the functional form of *β*(*t*) is mathematically derived and used to determine a sampling interval independent measurement that characterizes directionality.

### 2D Persistent Biased Random Walk Simulations and Data Fits

All simulation data were generated in MATLAB. They were fit using custom MATLAB software. This code is available for download online [27]. Data fits were calculated using the Levenburg-Marquardt least squared fitting algorithm [28] built into MATLAB.

### Migration Paths and Centroid Measurement Error

Differential interference contrast (DIC) image sequences capturing directionally migrating Polymorphonuclear Human Neutrophils were obtained from Ref. [17] along with cell centroid positions, **r**_*i*_(*t*_*n*_), where *i* is the migration path index and *t*_*n*_ is the time measured in multiples of the sampling interval, *t*_*n*_ = *n*Δ*t* (*n* = 1, 2, …). Centroid measurement error was estimated as follows. A cell was chosen at random and manually outlined five times. The five corresponding centroid positions were determined using the *regionprops* algorithm in MATLAB (the MathWorks; Natick, MA). The centroid measurement error of that cell, *σ*_*m*_, was calculated as the RMS displacement from the mean centroid position.

## Results

### Deriving the Directionality Time Model

When observing an ergodic, directionally biased random walk, there exists a sufficiently large sampling interval such that the motion will appear to be ballistic. Put in terms of log-log MSD slope, *β*(*τ*) transitions towards 2 as *τ* → ∞ for a directionally biased random walk. The idea of using log-log MSD slope to measure the transition time was recently proposed in a preceding article about neutrophil chemotaxis [17]. We suggested an empirical fit function for *β*(*t*) to quantify this time interval that we called directionality time. Here in this article, we rigorously develop the concept of directionality time from the bottom up by analytically deriving a *β*(*t*) fit function (without free parameters) and using biased and persistent random walk models to characterize its robustness. Directionality time is defined as the time scale above which motion appears ballistic (directional) and can be loosely interpreted as the time it takes for a random walker to orient towards an external cue.

To determine the mathematical meaning and robustness of directionality time, we analytically derive the log-log EASD slope, *β*(*t*), for three ergodic directionally biased random walk models:

1. Drift Diffusion (DD)
2. 2D Stepping Biased Random Walk (2D-SBRW)
3. 1D Persistent Biased Random Walk in Continuous Time (1D-PBRW)

These calculations are shown in detail in the supporting information (Appendix A in S1 File). The high level results are described here.

DD (model 1) was a suitable starting point because these processes are readily understood. For DD in *d* dimensions, with a diffusion constant *D*, and a drift speed *u*, the log-log EASD slope is shown in the supporting information (Eq. A2 in S1 File) to be

$$\begin{array}{c} \beta_{\text{DD}}\left(t\right)=\frac{1+\frac{2t}{t_{d}}}{1+\frac{t}{t_{d}}} \end{array}$$ (4)

where $t_{d}=\frac{2dD}{u^{2}}$ defines directionality time. Note that *β*(*t*) begins at *β*(0) = 1 and asymptotes towards 2. This is the signature of a directionally biased random walk. Directionality time is the time at which $\beta =\frac{3}{2}$ where the migration transitions from diffusive to directional. As *D* increases and/or *u* decreases, more observation time is required to determine that the motion is directionally biased.

Directional cell migration, though, is not a drift diffusion process. While drift may be an important factor when measuring the directionality of swimming cells, the process of cellular propulsion itself is better described kinematically by a 2D-SBRW (model 2). In a 2D-SBRW process, an object steps from one discrete position, **R**_*n*_, to the next, **R**_*n*+1_ (*n* = 0, 1, 2, …), such that the displacements between successive steps are biased towards a particular direction, ${\hat{\text{e}}}_{x}$ (Fig 1C). Notationally, all random variables are assigned capital letters. Using *L*_*n*_ and Θ_*n*_ to denote step lengths and polar angles (orientation with respect to ${\hat{\text{e}}}_{x}$) respectively, the stepwise EASD can be shown to be (see Ref. [25] for the derivation, and Eq. A5 in S1 File)

$$\begin{array}{c} \langle R_{n}^{2}\rangle =n\left\langle L^{2}\right\rangle +n\left(n-1\right){\left\langle L\cos\Theta \right\rangle }^{2}. \end{array}$$

The important information conveyed by this equation is that the motion is diffusive ($\langle R_{n}^{2}\rangle ∼n$) when *n* is small and directional ($\langle R_{n}^{2}\rangle ∼n^{2}$) when *n* is large. As before, the goal is to derive the directionality time (or number of steps) at which the motion transitions from diffusive to directional. By defining a constant instantaneous speed *v*, the approximation $n\approx \frac{vt}{\left\langle L\right\rangle }$ can be used to derive EASD as a function of time *t* instead of step number *n*. In the time representation, this is a model of a biased random walk (BRW) instead of an SBRW. Differentiating in log-log coordinates gives the log-log EASD slope (Eq. A8 in S1 File)

$$\begin{array}{c} \beta_{\text{BRW}}\left(t;\;t_{d}\right)=\frac{1+\frac{2t}{t_{d}}}{1+\frac{t}{t_{d}}} \end{array}$$ (5)

which is functionally identical to *β*_DD_ (Eq 4), now with directionality time given by (Eq. A9 in S1 File)

$$\begin{array}{c} t_{d}=\frac{\left\langle L\right\rangle }{v}(\frac{\left\langle L^{2}\right\rangle -{\left\langle L\cos\Theta \right\rangle }^{2}}{{\left\langle L\cos\Theta \right\rangle }^{2}}). \end{array}$$ (6)

The functional form of EASD slope is no different between models 1 and 2, except that the mathematical constants that constitute directionality time have changed. With Eq 5 holding true for all composite step length and directional bias distributions, this function can be used to measure the directionality time of many types of directionally biased motion. The equivalence between this form of directionality time and that derived for DD (below Eq 4) is shown in Appendix A (Eq. A12 in S1 File).

The generalized directionality time given in Eq 6 can be understood by considering the following example. Consider the case where the probability that a cell changes direction is constant from one moment to the next and that orientation angles Θ are chosen from a biased distribution independent of step lengths *L*. Then directionality time simplifies to (Eq. A13 in S1 File)

$$\begin{array}{c} t_{d}=t_{p}(\frac{2-c^{2}}{c^{2}}) \end{array}$$ (7)

where *c* ≡ ⟨cosΘ⟩, and *t*_*p*_ represents the reorientation time, the average time of each ballistic step. The term *c* is tangent-bias correlation (analogous to tangent-tangent correlation). Values of *c* ^2^ range from 0, corresponding to no orientation bias (PDF $\rho_{\Theta }(\theta )=\frac{1}{2\pi }$, where −*π* < *θ* ≤ *π*), to 1, corresponding to maximal orientation or anti-orientation bias (PDF *ρ*_Θ_(*θ*) = *δ*(*θ*) or *δ*(*θ* − *π*), where *δ* is the Dirac delta function). Directionality time depends only on the reorientation time and the extent to which the orientation is biased when a reorientation event occurs, increasing with the former and decreasing with the latter. In particular, the term $\frac{2-c^{2}}{c^{2}}$ ranges from 1 at maximal bias, to ∞ at no bias. It may appear odd that *t*_*d*_ → *t*_*p*_ (the equivalent of an average step duration) when the system is perfectly directional (*c* ^2^ → 1). However, this is no more than a subtlety of stepping random walks. There is no change in position defined at *t* < *t*_*p*_ because of the way continuous time was substituted in for discrete stepping number (*t* ≈ *nt*_*p*_). Therefore, the minimum time to determine that movement is directionally biased will always be greater than or equal to *t*_*p*_. No information can be gained from a random walker that has not yet taken any steps.

In the general case where *ρ*_*L*_ is not a Poissonian PDF but stepping time and directional bias are independent of one another, the constant 2 in the numerator of Eq 7 is replaced by $\frac{\left\langle L^{2}\right\rangle }{{\left\langle L\right\rangle }^{2}}$. Thus, the directionality time increases with increasing stepping time variance, as one would expect. This is relevant to processes such as Lévy Flights which correspond to a step length distribution with a long tail [29].

Since each step in the SBRW is accompanied by a memoryless reorientation, this model cannot be used to derive a log-log EASD slope equation that accounts for persistence. In order to consider the relation between directionality time and persistence, a continuous time random walk model must be derived, noted as the PBRW (model 3). This model is derived in 1D for simplicity using the biased telegrapher equation (Eq. A14 in S1 File) [24, 30]. To put this model in context, the unbiased telegrapher equation has been used to derive the dynamics and EASD of persistent random walks that describe the kinematics of chemokinesis [16, 31], as well as the motion of grasshoppers and kangaroos [24]. In the 1D-PBRW, an object moves with constant speed *v*, either left (−*x* direction) or right (+*x* direction) for some random run time (*T* ^(*l*)^ or *T* ^(*r*)^, respectively) before switching directions. Bias is induced by drawing left and right run times from nonequivalent distributions and characterized using tangent-bias correlation $c\equiv \langle \cos\Theta \rangle =\frac{\left\langle T^{\left(r\right)}\rangle -\langle T^{\left(l\right)}\right\rangle }{\left\langle T^{\left(r\right)}\rangle +\langle T^{\left(l\right)}\right\rangle }$ (*c.f*. Eq 7). The log-log EASD slope of the PBRW (Eq. A21 in S1 File) is more complicated than that for the BRW and DD because directionality over short time scales caused by persistence induces a zero-time log-log EASD slope *β*_PBRW_(0) = 2. As *t* increases and the orientational correlation of persistent motion is lost, *β*_PBRW_(*t*) dips towards 1. Except when *c* = 0, *β*_PBRW_(*t*) → 2 as *t* → ∞, as is the signature of directionally biased motion.

**Fig 2:**
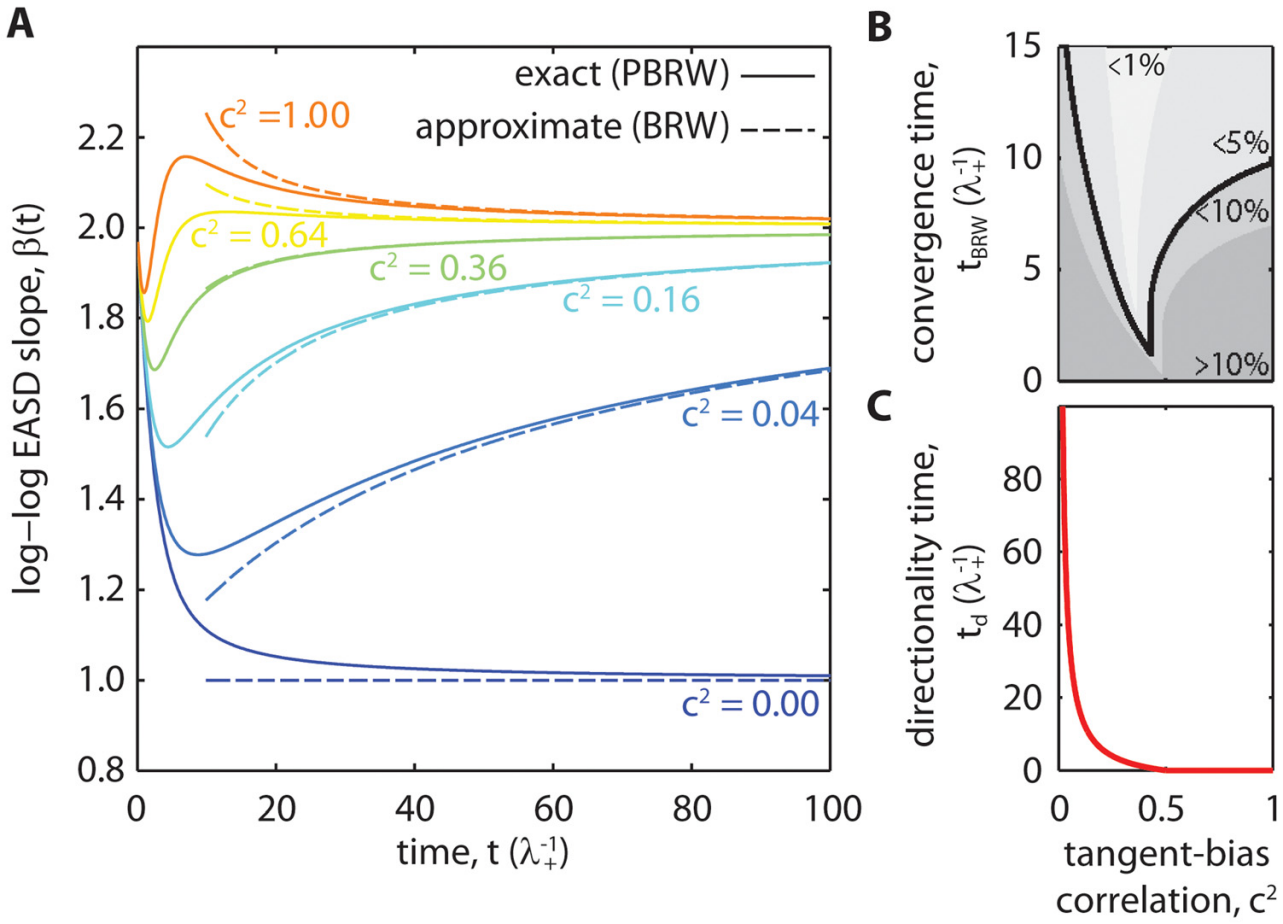
Log-log EASD slopes of the 1D-PBRW and its correspondence to the directionality time model. (A) Log-log EASD slopes of the 1D-PBRW model (*β*_PBRW_(*t*), solid curves) asymptotically approach the directionality time model (*β*_BRW_(*t*; *t*_*d*_), dashed curves) given a specific value of directionality time *t*_*d*_. These slopes are plotted against time for different values of the squared tangent-bias correlation, *c* ^2^ = ⟨cosΘ⟩^2^. In these plots, time is nondimensionalized by the persistence time-like parameter, $\lambda_{+}^{-1}$. Correlated orientation over short time scales gives *β*(0) = 2 regardless of directional bias. Except in the absence of directional bias (*i*.*e*. *c* ^2^ = 0), *β*_PBRW_(*t*) dips from 2 to 1, before asymptoting back toward 2 as *t* → ∞. (B) The convergence times above which the difference between *β*_PBRW_(*t*) and *β*_BRW_(*t*; *t*_*d*_) is less than 10, 5, and 1% are shown in gray scales plotted against *c* ^2^. The 5% convergence time, denoted *t*_BRW_ (black solid curve), is used for calculations in this work. (C) The corresponding directionality time plotted against *c* ^2^. The transition from random motion to directional motion $\left(\beta_{\text{BRW}}(t_{d})=\frac{3}{2}\right)$ does not occur in the domain, 0.5 ≤ *c* ^2^ ≤ 1. In this domain, directionality time is defined to be zero because the motion appears directional at all time scales.

These *β*_PBRW_(*t*) curves are plotted Fig 2A for multiple values of the tangent-bias correlation *c* ^2^ (solid curves). In this plot, time is in units of $\lambda_{+}^{-1}$, which is related to the average run time (persistence time). At sufficiently large time scales (Eq. A22 in S1 File)

$$\begin{array}{c} \beta_{\text{PBRW}}\left(t>t_{\text{BRW}}\right)≃\beta_{\text{BRW}}\left(t;\;t_{d}\right)=\frac{1+\frac{2t}{t_{d}}}{1+\frac{t}{t_{d}}} \end{array}$$ (8)

where *t*_BRW_ is the convergence time above which the difference between *β*_PBRW_ and *β*_BRW_ is less than 5% (Fig 2B). Therefore, directionality time can be measured by fitting *β* to *β*_BRW_(*t*; *t*_*d*_) at time scales larger than *t* = *t*_BRW_. The resulting measurement of *t*_*d*_ from this fit is given by (Eq. A23 in S1 File)

$$\begin{array}{c} t_{d}=\frac{1}{\lambda_{+}}\frac{2(1-2c^{2})}{c^{2}}. \end{array}$$ (9)

As with the BRW, *t*_*d*_ → ∞ for a random walk that is unbiased. When the bias is sufficient ($\frac{1}{2}\leq c^{2}\leq 1$), the gap between short time scale persistence and long time scale directionality vanishes such that the random walk appears directional at all time scales. By construction, we redefine negative values of directionality time to 0 in this domain to be consistent with the interpretation that directionality time is the observation time necessary to determine that motion is directional (Fig 2C).

The result *β*_DD_(*t*) = *β*_BRW_(*t*) ≃ *β*_PBRW_(*t*) at large time scales implies that one unique fit function can be used to measure *t*_*d*_ from any set of idealized migration path measurements. For brevity, we refer to this fit function as the *β*_BRW_-model. The *β*_BRW_-model is independent of the type of random walk process, returning values of *t*_*d*_ ranging from 0 when all time scales appear directional, to ∞ when motion is completely unbiased.

### 2D-PBRW and the Decoupling of Measurement Error

In the previous section, we calculated the log-log MSD slope for a 1D-PBRW (model 3) and showed that it was asymptotically equal to the *β*_BRW_-model. Here, we use computational Monte Carlo simulations to test if the *β*_BRW_-model holds in 2D in the same form that it was derived in 1D. We also demonstrate the robustness of the *β*_BRW_-model by adding 2D Gaussian noise (measurement error) to each sample of the centroid position and deriving a rule to decouple measurements of directionality time from this form of measurement error.

**Fig 3:**
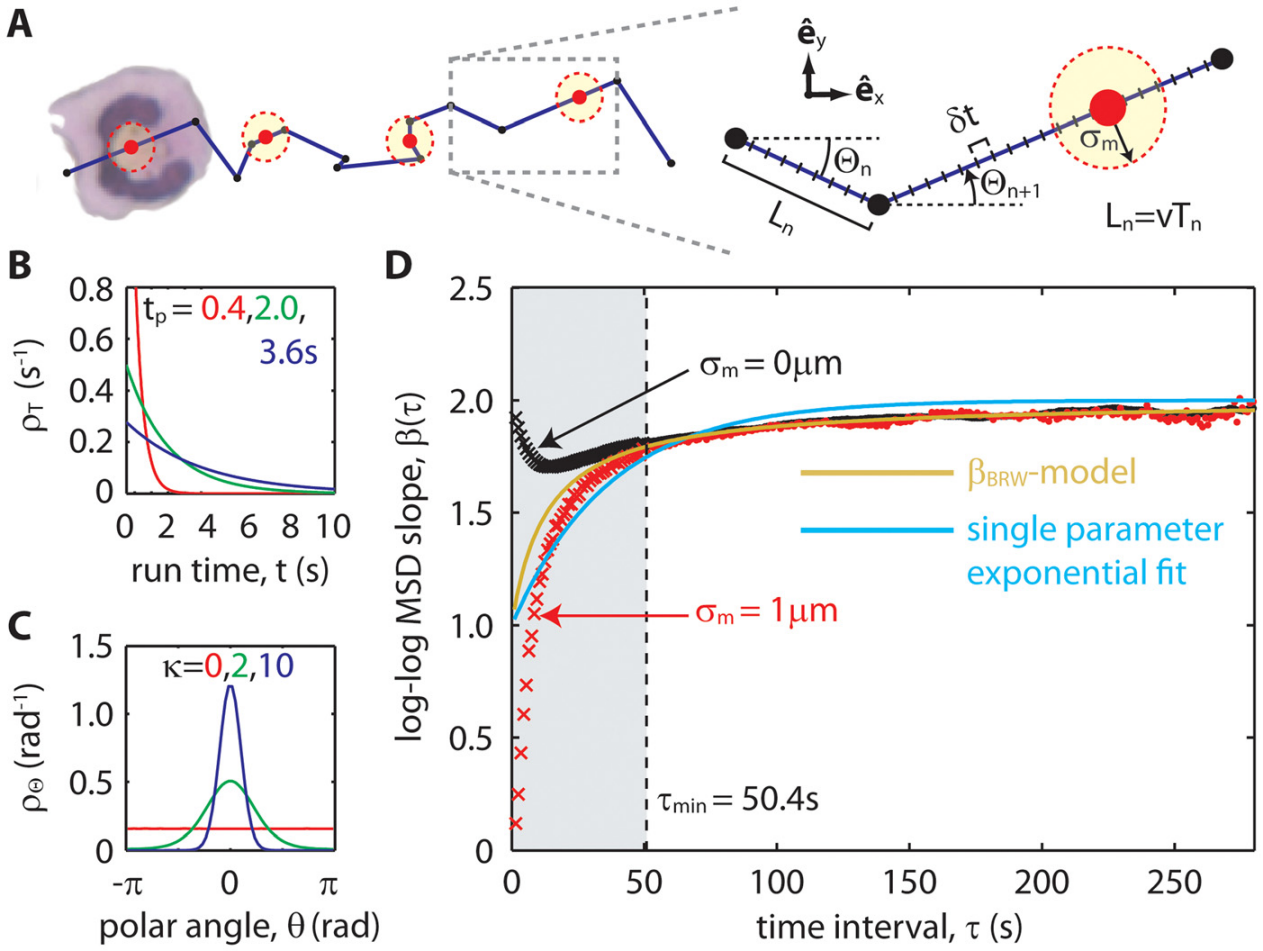
Measuring directionality time from simulated 2D-PBRW migration paths that resemble experimental data. (A) A schematic of the 2D-PBRW model and its corresponding random variables. A simulated random walker travels with constant speed *v* in a straight line with polar angle Θ_*n*_. After traveling in one direction for a run time *T*_*n*_, the random walker reorients and continues in a new direction, Θ_*n*+1_. For each random walk, positions were sampled in increments of sampling interval Δ*t*. Measurement error *σ*_*m*_ was added to each sampled position coordinate to generate migration paths resembling experimental data. (B) Run times, *T*_*n*_, were randomly selected from Poisson distributions with average run times of *t*_*p*_, known as the persistence time. (C) Polar angles, Θ_*n*_, were randomly selected from the von Mises distribution with bias factor *κ*. The value *κ* = 0 corresponds to unbiased motion. Increasing *κ* corresponds to more directional bias. (D) Log-log MSD slopes showing *β*(*τ*) for two values of centroid measurement error: *σ*_*m*_ = 0, 1 *μ*m (black and red, respectively; ×’s for *τ* < *τ*_min_ and •’s for *τ* > *τ*_min_; parameters *t*_*p*_ = 3.6 *s*, *κ* = 1.5, *v* = 0.3 *μ*m/s, Δ*t* = 1 *s*; ensemble sizes of *n* = 400). The minimum time interval above which the *β*_BRW_-model fit decouples from the effects of measurement error and persistence was estimated at *τ*_min_ = 50.4 *s*. Fitting the *β*_BRW_-model to data above this minimum time interval (green curve) gave virtually identical directionality times for both values of measurement error. Data in the *τ* < *τ*_min_ domain (×’s in the shaded region) were not used for fitting. A single parameter exponential fit function, $2-e^{-t/t_{d}}$ (cyan curve), also fit in the *τ* > *τ*_min_ domain, shows that a heuristic fit model does not accurately measure directionality time.

A diagram of the 2D-PBRW with measurement error is shown in Fig 3A. Continuous time was simulated in time steps of *δt*. Run times, *T*_*n*_ (times between reorientations), were drawn from Poisson distributions corresponding to persistence times *t*_*p*_, ranging from 0.4 to 3.6 s (Fig 3B). At the end of each run time, a reorientation occurred. Polar angles, Θ_*n*_, were drawn from von Mises distributions [32] centered about *θ* = 0 in the ${\hat{\text{e}}}_{x}$ direction. The von Mises distribution width, set by parameter *κ* (Fig 3C), was selected from values between 0 (uniform distribution) and 10 (relatively narrow Gaussian-like curve). The correspondence between *κ* and ⟨cosΘ⟩ is shown in S1 Fig. Positions along the 2D-PBRW were sampled periodically with sampling interval Δ*t* and 2D Gaussian noise with radius *σ*_*m*_ was added to each sampled position coordinate to resemble the measurement error of microscope tracked directionally migrating cells such as neutrophils [14, 17].

To investigate the effects of measurement error on directionality time, two ensembles of simulated trajectories were computed, one with *σ*_*m*_ = 0 and the other with *σ*_*m*_ = 1 *μ*m. These simulated trajectories were ergodic, thus allowing EASD and MSD to be swapped interchangeably (*β*(*t*) ↔ *β*(*τ*), with *t* ↔ *τ*). The corresponding measurements of *β*(*τ*) are shown Fig 3D (black and red, respectively). Visually, the deviations between the two datasets are greatest at short time scales (shaded region). Specifically, *β*(0) = 0 when *σ*_*m*_ > 0, instead of *β*(0) = 2 when *σ*_*m*_ = 0. Effectively, measurement error hides the identification of persistence in the short time scale limit.

Recall that *β*_PBRW_(*t*) converges to the *β*_BRW_-model above time *t*_BRW_ (see Eq 8 and Fig 2B). Measurement error sets an additional convergence time, *t*_*σ*_*m*__, above which the *β*_BRW_-model is valid. This time is derived analytically in the supporting information (Appendix A in S1 File, below Eq. A24; see also S2 Fig) by adding a measurement error term to the EASD of the PBRW model and calculating the time above which this modified PBRW model converges to within 5% of the *β*_BRW_-model:

$$\begin{array}{c} t_{\sigma_{m}}\approx 4.5\sqrt{2d}\frac{\sigma_{m}}{v_{\text{rms}}\left(\infty \right)}. \end{array}$$ (10)

Here, *d* is the number of dimensions, and *v*_rms_(∞) = lim_*τ* → ∞_ *v*_rms_(*τ*) = ∣*c*∣*v* is the root mean squared (RMS) speed asymptote, a measurable quantity that is sampling interval independent.

A minimum fit time, *τ*_min_, is defined as the larger of *t*_BRW_ and *t*_*σ*_*m*__ (Eq. A25 in S1 File). In this example, $\tau_{\text{min}}=t_{\sigma_{m}}\approx (4.5)(2)\frac{\left(1\mu \text{m}\right)}{(0.6)\left(0.3\mu \text{m/s}\right)}=50.4\text{s}\;$ (using Eq 10). Data in the *τ* > *τ*_min_ domain (Fig 3D, •’s) were fit to the *β*_BRW_-model (green curve) illustrating that the effects of measurement error and persistence can be decoupled from measures of directionality time by leaving out data below the time interval *τ*_min_. Although backwards extrapolation is required to measure directionality time in this example, it is not generally required (*i*.*e*. when *t*_*d*_ > *τ*_min_).

The possibility of using a heuristic model to measure directionality time is investigated by fitting the data in the *τ* > *τ*_min_ domain to a single parameter exponential function, $2-{\text{e}}^{-t/t_{d}}$ (Fig 3D, cyan curve). This model does not fit the data as well as the *β*_BRW_-model, returning overestimates of directionality time. Overall, measurements of directionality time using a heuristic model such as this generally satisfy the overall objective of measuring the time scale at which motion transitions from random to directional. However, lost when using a heuristic fit model are the consistency and interpretability of directionality time with respect to an analytical framework that characterizes directional motion.

Simulated migration paths corresponding to a range of parameter combinations (*t*_*p*_, *κ*, *σ*_*m*_) were analyzed to answer the following questions related to the robustness of the *β*_BRW_-model. For what type of motion does the *β*_BRW_-model fit well? When does it fail? And to what extent does directionality time decouple from measurement error by fitting at times *τ* > *τ*_min_? Goodness of fit was tested using the reduced chi-squared metric, $\chi_{\nu }^{2}$ (left column in S3A Fig). Fits labeled “good” (unshaded regions) were those with $\chi_{\nu }^{2}\leq 1$. All other fits were labeled “problematic” (yellow shaded regions). The *β*_BRW_-model fits were good over most of the parameter space. For non-directional motion (*κ* = 0), many of the *β*_BRW_-model fits were problematic, although the model still returned characteristically large directionality times. The *β*_BRW_-model fits were also problematic for directional motion with small *t*_*p*_ and *t*_*d*_ compared to the time scale of noise, *t*_*σ*_*m*__ (*i*.*e*. *t*_*p*_ and *t*_*d*_ ≪ *t*_*σ*_*m*__ corresponding to the bottom-right yellow regions in S3A Fig). Measurements of directionality time indicated that values of *t*_*d*_ increases slightly as *σ*_*m*_ increases (right column in S3A Fig). This weak monotonic coupling between *t*_*d*_ and *σ*_*m*_ occurs because measurement error decreases the value of *β*(*τ*) at all time scales (though most strongly at short time scales), thus slightly increasing the value of *t*_*d*_ obtained from fitting to the *β*_BRW_-model. This coupling was only significant at the lower limits of *t*_*p*_ (S3B Fig). Overall, these simulation results show that if motion is directional and *t*_*p*_ and *t*_*d*_ are not masked by the time scale of noise, *t*_*σ*_*m*__, then the *β*_BRW_-model fits robustly and yields a directionality time that is negligibly skewed by measurement error.

For comparison, the sampling interval dependent metrics (TAD, TAD persistence, tortuosity, and tangent-tangent correlation) were calculated for the simulation trajectories (S4 Fig). As expected, the numerical values associated with these metrics varied significantly with sampling interval, coupling strongly to measurement errors at the smaller sampling intervals.

### Application to Real Data

Above, we have built the framework for the *β*_BRW_-model that characterizes idealized biased random walks and decouples from measurement error. With this framework in place, we consider complexities in experimental data that may cause deviations from the idealized *β*_BRW_-model. The primary causes of deviation can be grouped into three categories:

1. Persistence—correlated orientation below a corresponding persistence time
2. Position Variance—caused by measurement error and/or parametric variance across the ensemble (*i.e*. population heterogeneity)
3. Nonergodicity—occurs when the parameters of motion change over time

Methods for handling these deviations are addressed below.

Deviations caused by persistence and measurement error induced position variance can be decoupled from the *β*_BRW_-model by fitting above the independently calculated minimum fit time *τ*_min_ discussed above (*c.f*. Fig 3D). That leaves deviations caused by parametric variance and nonergodicity to be addressed.

Parametric variance, denoted $\sigma_{p}^{2}$, is the variance in distance traveled caused by variances of the random walk parameters across the ensemble (*i.e*. population heterogeneity). For example, the variance in distance traveled at long time scales due to a spread in the instantaneous speed parameter, *δv*, is $\sigma_{p}^{2}=c^{2}{\left(\delta v\right)}^{2}t^{2}$. Parametric variance systematically increases measurements of EASD and is usually the dominant cause of deviation between *β*(*t*) and the *β*_BRW_-model at large time scales (see Eq. B2). Deviations caused by parametric variance cannot be corrected by fitting above a minimum fit time, *τ*_min_.

Ergodicity is the conversion factor that maps EASD to TASD, given in Eq 3. When a process is ergodic, EASD and MSD are interchangeable, and so are calculations of *β* based on either EASD or MSD. When a process is nonergodic, for example when the instantaneous migration speed changes significantly over time across the entire ensemble, measurements of *β*(*τ*) based on MSD will deviate from the *β*_BRW_-model which is derived from the EASD. Deviations caused by ergodicity occur at all time scales and, like deviations caused by parametric variance, cannot be corrected for by fitting above a minimum fit time, *τ*_min_.

We show in Appendix B (Eq. B9 in S1 File) that log-log MSD slope *β*(*τ*) can be decomposed into the *β*_BRW_-model plus two other terms that correct for deviations caused by position variance and nonergodicity: *β*(*τ*) = *β*_BRW_(*τ*)+*β*_*σ*_(*τ*)+*β*_*ξ*_(*τ*). The term *β*_*σ*_ accounts for deviations caused by position variance (primarily parametric variance), and *β*_*ξ*_ accounts for deviations caused by nonergodicity. The position variance correction is calculated by using experimentally observed variances in distance traveled to estimate how much parametric variance and measurement error have an effect on MSD, and the corresponding log-log MSD slope (Eq. B10 in S1 File). The nonergodicity correction can also be calculated from experimental data (Eq. B11 in S1 File) but the result is often relatively noisy because time averaging cannot be applied to reduce statistical noise. To get a better signal to noise on measurements of *β*_*ξ*_(*τ*), the correction can instead be calculated by simulating biased random walks using the experimentally observed speed distribution. The simulation of these trajectories is repeated until a sufficient signal to noise is achieved for the measurement of *β*_*ξ*_(*τ*). A recipe for these simulations can be found in the supporting information (Appendix B in S1 File) with sample code online [27]. In many cases, however, simulations are unnecessary because nonergodic migration data can be made nearly ergodic by choosing to analyze truncated migration segments over which the instantaneous speed remains relatively constant. We recommend the latter approach whenever possible.

### Neutrophil Chemotaxis Example

**Fig 4:**
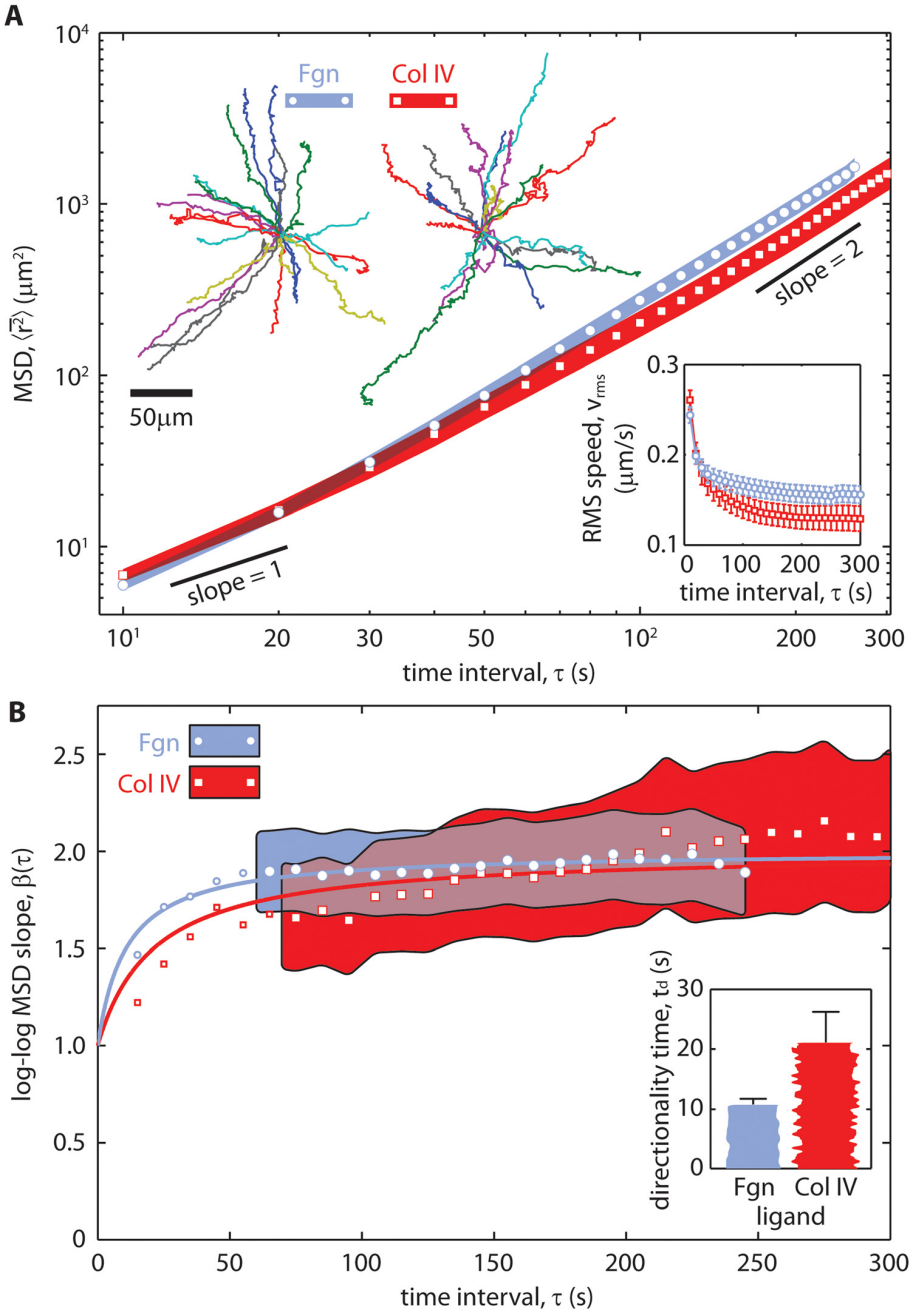
Measuring directionality time from experimental data. (A) The observed mean squared displacements (MSDs) of chemotactic human Polymorphonuclear Neutrophils (PMNs) migrating on fibrinogen (Fgn, blue, circles, n = 25) and collagen IV (Col IV, red, squares, n = 19) coated polyacrylamide gels with a Young’s modulus of 10 kPa. The shaded regions indicate the standard errors of the MSD. The corresponding trajectories (top-left) and RMS speed (bottom-right) are also shown. The RMS speed asymptotes, *v*_rms_(∞), for Fgn and Col IV are approximately 0.15 and 0.13 *μ*m/s, respectively. (B) Log-log MSD slopes corrected for position variance, *β* − *β*_*σ*_, are plotted against time interval *τ* and fit to the *β*_BRW_-model (solid curves) to obtain directionality times, *t*_*d*_. The shaded regions indicate standard deviations on log-log MSD slope at time intervals above the minimum fit time. The resulting directionality times are depicted in the bar graph to the bottom-right, with error bars corresponding to 68% confidence intervals.

In this section, an implementation of the *β*_BRW_-model to measure directionality time is demonstrated on migration paths of chemotactic human polymorphonuclear neutrophils (PMNs) following the step-by-step procedure outlined in S5 Fig. PMN migration paths, **r**(*t*), were obtained from data provided by O’Brien *et al*. [17], and the corresponding centroid measurement errors were calculated as described in the methods section. Two sets of data were analyzed, each containing the trajectories of chemotactic PMNs migrating on the 2D surfaces of polyacrylamide gels (Young’s modulus: 10 kPa) towards a source of the chemoattractant *formyl-methionyl-leucyl-phenylalanine*. The difference between the two data sets was the coating on the gel surface: one was human fibrinogen (Fgn), the other was human type IV collagen (Col IV). Tangent-tangent correlation measurements gave a persistence time upper bound of 5 s (S6C Fig). Note, PMN migration persistence decreases significantly as surface stiffness decreases below 100 kPa [14–16]. PMN persistence times on stiffer surfaces can be much larger. EASD was calculated but was noisy. Therefore, MSD was also calculated to reduce the statistical noise (Fig 4A).

Data was checked for nonergodicity by measuring the ensemble averaged instantaneous speed (EAIS) over time. Non constant EAIS is an indicator of nonergodic motion because it signifies that the underlying characteristics of motion change over time and that the time averaged squared displacements will be different from the ensemble averaged squared displacements. EAIS was not constant over all time but was relatively constant over the first 400 s of migration. Therefore, all long nonergodic trajectories were truncated at 400 s (40 centroid measurements each) leaving an ensemble of ergodic trajectories from which directionality time could be measured. If EAIS were still changing significantly in time, then the ergodicity correction term *β*_*ξ*_ could have been calculated using the recipe in the supporting information (Appendix B in S1 File).

Next, *v*_rms_(∞) was calculated (Fig 4A, bottom-right inset): *v*_rms_(∞) ≈ 0.15 and 0.13 *μ*m/s for Fgn and Col IV, respectively. Taken together with the upper limit on *t*_*p*_, the minimum fit time was calculated using Eq 10 for both data sets: *τ*_min_ ≈ 60 s and 69 s for Fgn and Col IV, respectively. With these data, the minimum fit times corresponded to the time scale below which measurement error skewed the data. The variance corrected log-log MSD slope, *β* − *β*_*σ*_ (see Fig 4B), was fit to the *β*_BRW_-model at time intervals *τ* > *τ*_min_ (regions with shaded error bars). The resulting directionality times were 10.7 ± 1.0 s and 21.2±5.1 s for Fgn and Col IV, respectively (Fig 4B, bottom-right inset). With 19 PMN trajectories in the Col IV data set, compared to 25 in the Fgn data set, the Col IV fit was noisier. The uncertainty on these measurements of directionality time was relatively large because both values fell below the minimum fit time. More precise values of directionality time could be acquired if the measurement errors were smaller or the directionality times were larger. For comparison, the sampling interval dependent metrics (TAD, TAD persistence, tortuosity, and tangent-tangent correlation) were calculated for this data (S6 Fig). The numerical values of these measurements varied significantly with sampling interval and their coupling to measurement errors were unknown.

### Summary

We have completed a three part description of directionality time, including an analytical derivation, computational simulations to investigate robustness, and an application to noisy real world ensembles of the directional migration paths of chemotactic neutrophils. In comparison to the sampling interval dependent metrics (see S4 and S6 Figs), the directionality time metric is nearly independent of parameters that are constrained by the experimental apparatus and/or chosen arbitrarily by humans. Whereas speed and persistence time are sufficient for characterizing non directional migration, one additional metric, directionality time, along with speed and persistence time, suffices to characterize directional migration.

## Supporting Information

S1 File Supporting Text. This supporting text is divided into three sections: A mathematical notation guide; Appendix A: Analytical modeling of directional motion; and Appendix B: Deviations caused by variances and nonergodicity.
(PDF)

S1 Fig Tangent-bias correlation, *c* = ⟨cosΘ⟩, plotted against the von Mises bias factor, *κ*. This curve shows the correspondence between *c* used in the analytically derived biased random walk models, and *κ* used in the simulated biased random walk model. This curve was calculated from the definition of the von Mises distribution. Specifically, $\langle \cos\Theta \rangle =\frac{I_{1}(\kappa )}{I_{0}(\kappa )}$ where *I*_*n*_ is the modified Bessel function of the first kind, order *n*.
(EPS)

S2 Fig Time scales at which *β*_PBRW_(*t*) converges to the *β*_BRW_-model when measurement error is nonzero (*σ*_*m*_ > 0). Times above which the difference between *β*_PBRW_(*t*) and the *β*_BRW_-model is less than 5% are shown as solid curves correspond to values of the constant $\epsilon =\frac{\sqrt{2d}\sigma_{m}\lambda_{+}}{v}$, which is proportional to measurement error. The convergence time is estimated by the equation $\lambda_{+}t_{\sigma_{m}}\approx \frac{4.5\epsilon }{c}$, as derived in the supporting information (Appendix A in S1 File, below Eq. A24). When implementing a *β*_BRW_-model fit to measure directionality time, *β*(*τ*) curves are fit at time intervals *τ* > *t*_*σ*_*m*__ to decouple directionality time from measurement error.
(EPS)

S3 Fig Additional data plots investigating the robustness of the *β*_BRW_-model. (A) Goodness of fit and directionality time measurements across a range of parameter combinations (same parameters as in Fig 3D except that *t*_*p*_, *κ*, and *σ*_*m*_ all vary, and Δ*t* = 10 *s*). Squares on phase diagrams indicate parameter combinations where ensembles of random walks were simulated (*n* = 4000 migrations per ensemble). Goodness of fit was measured by calculating reduced chi squared, $\chi_{\nu }^{2}=\frac{1}{\nu }\sum_{n}\frac{{\left(\beta (\tau_{n})-\beta_{\text{BRW}}(\tau_{n};t_{d})\right)}^{2}}{\sigma_{\beta }^{2}(\tau_{n})}$, where *σ*_*β*_ is the standard deviation (spread) on values of *β*(*τ*_*n*_) used for fitting, and *ν* is the degrees of freedom. A fit was categorized as “good” if $\chi_{\nu }^{2}\leq 1$, otherwise it was categorized as “problematic.” The directionality time model worked robustly except near *κ* = 0 and when *t*_*p*_ and *t*_*d*_ ≪ *τ*_min_. The latter is the limit where measurements of directionality time can no longer be resolved (problematic fits at low *t*_*p*_, high *σ*_*m*_, as *κ* increases beyond 6 where *t*_*d*_ is effectively negligible). (B) Directional migration efficiency is inversely proportional to directionality time (in units of *t*_*p*_). Directional migration efficiency is defined as the ensemble averaged distance traveled ⟨*R*(*t*)⟩ divided by the distance, *vt*, a ballistic walker would have traveled if it had the same speed. This plot, calculated for the values of *κ* indicated, shows that *t*_*d*_ is a proxy for the distance a walker will travel, and that *t*_*d*_ best decouples from measurement error as persistence time *t*_*p*_ increases. Note that random walks corresponding to *κ* > 10 are nearly ballistic (*t*_*d*_ ≈ 0) and there are no significant changes in dynamics as *κ* → ∞.
(EPS)

S4 Fig Sampling interval dependent metrics applied to simulation data (2D-PBRW, *t*_*p*_ = 3.6 s, *κ* = 1.5, and *v* = 0.3 *μ*m/s). The position of the random walk was sampled every Δ*t* = 1, 4, or 20 *s*, with sampling errors of *σ*_*m*_ = 0 (blue bars and thin blue curves) or 0.1 *μ*m (green bars and thick green curves). (A) Ensemble averaged turning angle distributions (TAD) based on sampling intervals of 1 *s* (solid curves) and 4 *s* (short-dashed curves). TAD persistence is the fraction of all turning angles between $\pm \frac{\pi }{2}$ (inset, error bars are standard error of the ensemble mean). When *σ*_*m*_ = 0, TAD persistence is smallest when Δ*t* ≈ *t*_*p*_. This is time scale of reorientation and consequently the sampling interval with which the motion appears most random. Small amounts of measurement error (*σ*_*m*_ = 0.1 *μ*m) hide the persistence of motion that would otherwise be measured at the smallest sampling interval Δ*t* = 1 s. These data show that TAD persistence is sampling interval dependent. (B) Ensemble averaged tortuosity (error bars are standard error of the ensemble mean). As with TAD persistence, tortuosity is sampling interval dependent, increasing with Δ*t*. (C) Tangent-tangent correlation curves. Tangents are calculated based on the forward displacement between nearest sampled points. Therefore, tangent-tangent correlation is sampling interval dependent. In particular, randomness at short time scales are not resolved as Δ*t* increases. The persistence time, *t*_*p*_, can be back-measured from the tangent-tangent correlation curves if there is sufficient temporal resolution (Δ*t* ≤ *t*_*p*_). Each of these metrics are sampling interval dependent and couple to measurement error at short sampling intervals. Hence, these metrics are not generally comparable from one experiment to the next.
(EPS)

S5 Fig Step-by-step flow-chart for processing experimental migration data to measure directionality time. (EPS)

S6 Fig Sampling interval dependent metrics applied to chemotactic polymorphonuclear neutrophils (PMNs) on fibrinogen (Fgn, blue colors) and human collagen IV (Col IV, red colors) coated polyacrylamide gels of elastic modulus 10 kPa. (A) Ensemble averaged turning angle distributions (TAD) are plotted based on measurements of turning angles at time intervals of 10 s (solid curves) and 60 s (dashed curves). TAD persistence, calculated as the fraction of all turning angles between $\pm \frac{\pi }{2}$, is shown in the inset (error bars are standard error of the ensemble mean). (B) Ensemble averaged tortuosity measured at the same sampling intervals (error bars are standard error of the ensemble mean). (C) Tangent-tangent correlation curves also measured at sampling intervals Δ*t* = 10 and 60 *s*. Tangents are calculated based on the forward displacement between nearest sampled points. Therefore tangent-tangent correlation curves show more correlation when calculated at the 60 *s* sampling interval, compared to the 10 *s* interval. For both Fgn and Col IV, tangent-tangent correlation curves drop towards their asymptote at a persistence time of *t*_*p*_ < 10 s. Regardless of the sampling interval, chemotaxis on Fgn is more correlated than chemotaxis on Col IV, a result that is consistent with our measurements of directionality time. Each of these metrics are sampling interval dependent and couple to measurement error. Hence, these metrics are not generally comparable from one experiment to the next.
(EPS)
