# Supplementary material for: Describing Directional Cell Migration with a Characteristic Directionality Time
Source: PLoS One. 2015 May 20;10(5):e0127425. doi: 10.1371/journal.pone.0127425 (PMC4439174; doi:10.1371/journal.pone.0127425)
Supplement: S1 File — This supporting text is divided into three sections: A mathematical notation guide; Appendix A: Analytical modeling of directional motion; and Appendix B: Deviations caused by variances and nonergodicity. (PDF) [file pone.0127425.s001.pdf]

Mathematical Notation Guide, Appendix A, and Appendix B for "Describing Directional Cell Migration with a Characteristic Directionality Time"

Alex J. Loosley[1], Xian M. O'Brien[2], Jonathan S. Reichner[2], Jay X. Tang[1,*]

1 Department of Physics, Brown University, Providence, RI, USA

2 Division of Surgical Research, Department of Surgery, Rhode Island Hospital and the Warren Alpert Medical School of Brown University, Providence, RI, USA

∗ Corresponding author

E-mail: Jay_Tang@brown.edu (JXT)

Mathematical Notation

notation	usage
δt	simulation time step
Δt	sampling interval
t	time
τ	time interval
**bold** variables	vectors
CAPITALIZED variables	random variables
$\rho_{T}(t)$	probability density function (PDF),<br>(the probability of measuring random variable T between the values t and $t + \mathrm{d}t$)
$\mathbf{R}$	random variable for position used in analytical models<br>(corresponding to the PDF, $\rho_{\mathbf{R}}(\mathbf{r})$)
Θ	random variable for 2D orientation<br>(the polar angle of the instantaneous velocity vector)
$\overline{\text{overline}}$	average over time t
$\langle\rangle$	average over ensemble or analytical PDF
$c = \langle\cos\Theta\rangle$	tangent-bias correlation (averaged over $\rho_{\Theta}(\theta)$)
$\mathbf{r}$	experimentally measured position
$\mathbf{v}$	instantaneous velocity
v_{rms}	root mean squared speed
ϕ	turning angle (not to be confused with polar angle θ)
$\overline{\hat{\mathbf{v}}(t+\tau)\cdot\hat{\mathbf{v}}(t)}$	tangent-tangent correlation
t_p	persistence time
t_d	directionality time

Appendix A: Directionality Time Derived from Analytical Models of Directional Motion

Introduction and Notation

In this appendix, three random walk models are discussed. Positions, $\mathbf{R}$, are specified by a two dimensional (2D) Cartesian coordinate system with unit vectors $\hat{\mathbf{e}}_x$ and $\hat{\mathbf{e}}_y$ (Fig. 1 C). The orientation of movement is described by polar angle θ ($\pi < \theta \leq \pi$) defined with respect to the positive x-axis. The random walker begins by moving with speed V_1 in a particular direction Θ_1 for a particular time T_1, before reorienting and moving with a new speed V_2, in a new direction Θ_2, for another time period given by T_2. This process continues and is altogether described by speeds V_n, angles Θ_n, and step durations T_n ($n = 1, 2, ...$), which are random values of speed v, polar angle θ, and time t, respectively. Each random variable is associated with a probability density function (PDF), $\rho_{_V}(v)$, $\rho_{_\Theta}(\theta)$, and $\rho_{_T}(t)$. For each random walk model described in this appendix (SI Appendix A), we define the relevant variables, parameters, and distributions, and use these to determine the ensemble averaged squared displacement (EASD), $\langle R^2(t)\rangle$, and log-log EASD slope, $\beta(t)$. From $\beta(t)$, we derive the model specific meaning of directionality time, t_d, the measure that is central to this work. A universal, biased random walk model invariant fit function is derived to measure t_d from EASD data.

Model 1: Drift Diffusion (DD)

A walker undergoing a Diffusion in 2D (without drift) obeys the following rules:

1. **Stepwise Position**: The position after n steps is given by the random variable $\mathbf{R}_n = \sum_{j=1}^{n} V_j T_j \left[\cos(\Theta_j)\hat{\mathbf{e}}_x + \sin(\Theta_j)\hat{\mathbf{e}}_y\right]$.
2. **Constant step duration**: $\rho_{_T}(t) = \delta(t - \lambda^{-1})$, *i.e.* $T_j = \lambda^{-1}$ for all j
3. **No directional bias**: $\rho_\Theta(\theta) = \frac{1}{2\pi}$ where $-\pi < \theta \leq \pi$
4. **Constant track speed**: $V_j = v$

where $\delta(...)$ is the Dirac delta function, λ is the reorientation frequency, and the other terms are defined in the Appendix A Introduction. The notion for average step duration, λ^{-1}, is used for consistency with the models below. The addition of drift to a diffusion process adds an additional term to the stepwise

position equation. Without loss of generality, one can define a drift velocity along the x-axis, $\mathbf{u} = u\,\hat{\mathbf{e}}_x$. Subsequently, Rule 1 would be redefined as

1'. **Stepwise Position**: The position after n steps is given by the random variable $\mathbf{R}_n = \sum_{j=1}^{n} \frac{v}{\lambda} \left[\cos(\Theta_j)\hat{\mathbf{e}}_x + \sin(\Theta_j)\hat{\mathbf{e}}_y\right] + \frac{nu}{\lambda}\hat{\mathbf{e}}_x$.

One example of DD would be the motion of an object trapped at an air-water interface in the presence of a surface current (air or water). If the object were charged, drift could also be induced by applying a uniform electric field (*i.e.* carrier drift) [1].

The ensemble averaged squared displacement (EASD) of a DD in d dimensions has been shown to be [2]

$$\left\langle R^2(t)\right\rangle = 2dDt + u^2t^2 \tag{A1}$$

where $D \equiv \frac{v^2}{\lambda}$ is the diffusion constant. At large times, motion is directional ($\langle R^2(t)\rangle \rightarrow u^2t^2 \sim t^2$) with a root mean squared (RMS) speed, $v_{\mathrm{rms}}(t) = \frac{\sqrt{\langle R^2(t)\rangle}}{t}$, that asymptotes to $v_{\mathrm{rms}}(\infty) = u$. Differentiation in log-log coordinates gives the log-log EASD slope

$$\beta_{\mathrm{DD}}(t;\, t_0) = \frac{1 + 2\frac{t}{t_0}}{1 + \frac{t}{t_0}} \tag{A2}$$

where time constant, $t_0 = \frac{2dD}{u^2}$, incorporates both model parameters D and u. Defining directionality time as the time when $\beta = \frac{3}{2}$ provides a time scale where the migration transitions from diffusive to directionally drifting. This definition gives $t_d = t_0$ or

$$t_d = \frac{2dD}{u^2}. \tag{A3}$$

The greater the diffusion constant and/or the lower the drift velocity, the more time is required to observe a directional bias.

Model 2: 2-D Stepping Biased Random Walk (2D-SBRW)

A walker undergoing a 2-D Stepping Biased Random Walk (2D-SBRW) obeys the following rules:

1. **Stepwise position**: The position after n steps is given by the random variable $\mathbf{R}_n = \sum_{j=1}^{n} L_j \left[\cos(\Theta_j)\hat{\mathbf{e}}_x + \sin(\Theta_j)\hat{\mathbf{e}}_y\right]$.

2. **Random step length**: L_j is drawn from a distribution with PDF, $\rho_L(l)$.
3. **Directional Bias**: Θ_j is drawn from a circular distribution (*i.e.* von Mises, Wrapped Normal, *etc.* [3]) with PDF $\rho_\Theta(\theta)$ that is symmetric about $\theta = 0$ ($-\pi < \theta \leq \pi$).
4. **Constant track speed**: $V_i = v$

It can be shown that the EASD of a 2D-SBRW after n steps is given by

$$\left\langle R_n^2 \right\rangle = n \left\langle L^2 \right\rangle + n(n-1) \left\langle L \cos\Theta \right\rangle^2 + \left\langle L \sin\Theta \right\rangle^2 \tag{A4}$$

(see Ref. [4] for a derivation). With a directional bias PDF that is symmetric about $\theta = 0$ (*c.f.* rule 3), $\langle \sin\Theta \rangle = 0$ and the EASD simplifies to

$$\left\langle R_n^2 \right\rangle = n \left\langle L^2 \right\rangle + n(n-1) \langle L \cos\Theta \rangle^2. \tag{A5}$$

Note that the motion is diffusive when n is small ($\langle R_n^2 \rangle \sim n$) and directional when n is large ($\langle R_n^2 \rangle \simeq n^2 \langle L \cos\Theta \rangle^2$). The proportionality constant of directional motion, $\langle L \cos\Theta \rangle^2$, is nonzero if and only if the combined PDF governing L and Θ generates directional motion. This term will be discussed in detail below. Also note that this step by step random walk has no persistent directionality at low n because the direction of motion changes with every step.

In order to calculate log-log EASD slope, $\beta(t)$, an average track speed v is defined and the relation $n \approx \frac{vt}{\langle L \rangle}$ is used to transform $\langle R_n^2 \rangle \to \langle R^2(t) \rangle$. In the general time representation, this is a model of a biased random walk (BRW):

$$\left\langle R^2(t) \right\rangle = \frac{vt}{\langle L \rangle} \left[\left(\langle L^2 \rangle - \langle L \cos\Theta \rangle^2 \right) + \frac{vt}{\langle L \rangle} \langle L \cos\Theta \rangle^2 \right] \tag{A6}$$

with an RMS speed that asymptotes to

$$v_{\mathrm{rms}}(\infty) = \frac{\langle L \cos\Theta \rangle}{\langle L \rangle} v. \tag{A7}$$

Differentiating in log-log coordinates gives the log-log EASD slope

$$\beta_{\mathrm{BRW}}(t;\, t_0) = \frac{1 + \frac{2t}{t_0}}{1 + \frac{t}{t_0}} \tag{A8}$$

where $t_0 = \frac{\langle L \rangle}{v}\left(\frac{\langle L^2\rangle - \langle L\cos\Theta\rangle^2}{\langle L\cos\Theta\rangle^2}\right)$. Note that β_{BRW} has the same functional form as β_{DD} (Eq. A2). Only the mathematical constants have changed. Analogous to the DD treatment, defining directionality time at the transition point, $\beta = \frac{3}{2}$, gives $t_d = t_0$, or

$$t_d = \frac{\langle L \rangle}{v}\left(\frac{\langle L^2\rangle - \langle L\cos\Theta\rangle^2}{\langle L\cos\Theta\rangle^2}\right). \tag{A9}$$

This result simplifies further when the orientation angles $\{\Theta_j\}$ are selected independent of the step lengths $\{L_j\}$. Thus $\langle L\cos\Theta\rangle = \langle L\rangle c$, where $c = \langle\cos\Theta\rangle$ is called tangent-bias correlation because it describes the correlation of the random walk orientation (tangent vectors) to the direction of bias ($\theta = 0$). Squared tangent-bias correlation, c^2, ranges from 0, corresponding to no orientational bias $\left(\rho_\Theta(\theta) = \frac{1}{2\pi} \text{ where } -\pi < \theta \leq \pi\right)$, to 1, corresponding to maximal orientational bias ($\rho_\Theta(\theta) = \delta(\theta)$ or $\delta(\theta - \pi)$). Using this result, the asymptotic RMS speed (Eq. A7) simplifies to

$$v_{\mathrm{rms}} = |c|v \tag{A10}$$

and the directionality time becomes

$$t_d = \langle T \rangle \left(\frac{\frac{\langle T^2\rangle}{\langle T\rangle^2} - c^2}{c^2}\right). \tag{A11}$$

This generalized directionality time equation is more easily understood by considering two examples. The first example shows the equivalence between the directionality time equations derived for DD and the 2D-SBRW. Consider the case of a drift diffusion-like process where $\langle T\rangle^2 = \langle T^2\rangle = \delta t^2$ and the polar angle PDF is $\rho_\Theta(\theta) = \frac{1}{2\pi}\left[1 + \frac{u}{v}\cos(\theta)\right]$ ($u \leq v$, where u is drift speed and v is stepping speed). This gives a tangent-bias correlation of $c = \frac{u}{2v}$, and directionality time: $t_d = \frac{\delta t}{u^2}\left[4v^2 + u^2\right]$, where δt is the stepping time. Rewriting the instantaneous speed as $v = \frac{\delta l}{\delta t}$ where δl is the step length, and taking the continuum limit (δt, $\delta l \to 0$) gives

$$t_d = \frac{1}{u^2} \lim_{\delta t, \delta l \to 0}\left[\frac{4\delta l^2}{\delta t} + u^2 \delta t\right]. \tag{A12}$$

Taking the limit of the first term is the mathematical definition of the diffusion constant, D [5]. Taking the limit of the second term gives 0, leaving $t_d \propto \frac{D}{u^2}$. Up to a proportionality factor that appears because we did not model DD with an exact PDF, Eq. A12 shows the equivalence between Eq. A11 and Eq. A3,

and that directionality time depends only on the amount of diffusivity (D), and the external parameter (or more generally, the set of external parameters) that drive directional bias (u).

The second example is a case with a variable stepping time. Consider the case where the probability of measuring a step length T between t and $t+\mathrm{d}t$ is given by a Poissonian PDF, $\rho_T(t) = \frac{1}{t_p}\mathrm{e}^{-\frac{t}{t_p}}$. Then, $\langle T\rangle = t_p$, $\langle T^2\rangle = 2t_p^2$, and directionality time simplifies to

$$t_d = t_p\left(\frac{2-c^2}{c^2}\right). \tag{A13}$$

Importantly, directionality time is independent of speed v. It depends only on the average step duration t_p and the extent to which the orientation is biased when a reorientation occurs (given by c^2). Directionality time decreases when either the reorientation rate ($1/t_p$) or orientational bias (c^2) increase. In particular, the term $\frac{2-c^2}{c^2}$ scales from 1 at maximal bias, to ∞ at no bias. It may appear odd that $t_d \to t_p$ (the average time of one step) when the system is perfectly directional ($c \to 0$). However, this is no more than a subtlety of stepping random walks. Based on the way EASD was converted from the stepping number domain to the continuous time domain, positions are only allowed to change in increments of $\langle T\rangle = t_p$. Therefore, the minimum time to determine movement is directionally biased will always be greater than or equal to t_p. No information can be gained from a random walker that has not yet taken any steps. Since each step is accompanied by reorientation, this model cannot be used to derive a log-log EASD slope equation that accounts for short time scale directionality known as persistence. In order to consider the relation between directionality time and persistence, we consider a continuous time random walk model in the following section.

Model 3: 1-D Persistent Biased Random Walk (1D-PBRW) in Continuous Time

A walker undergoing a 1-D Persistent Biased Random Walk (1D-PBRW) obeys the following rules:

1. **Stepwise position**: The position after n steps is given by the random variable $X_n = \left(\sum_{j=(odd)}^{n} V_j T_j^{(r)} - \sum_{i=(even)}^{n} V_j T_j^{(l)}\right)$, where l and r indicate movement to the left (-x direction) and right (+ x direction), respectively.[1]

[1] Although the stepwise position is useful for introducing the model, below we derive the position distribution in continuous time.

2. **Variable leftward and rightward step durations**: The leftward and rightward durations are drawn from Poissonian PDFs: $\rho_{T^{(k)}}(t) = \lambda_k e^{-\lambda_k t}$, where $k = l, r$.
3. **Directional Bias**: This is set by the difference between the average rightward and leftward step durations in rule 2: $c \equiv \langle \cos\Theta \rangle = \frac{\langle T^{(r)} \rangle - \langle T^{(l)} \rangle}{\langle T^{(r)} \rangle + \langle T^{(l)} \rangle} = \frac{\lambda_l - \lambda_r}{\lambda_l + \lambda_r}$.
4. **Constant track speed**: $V_j = v$

The 1D-PBRW walker alternates between two possible directional states, traveling left (l) or right (r). To allow for persistence, a PDF for the position of the PBRW object, $\rho_X(x, t)$, is derived in continuous time from a partial differential equation (as opposed to from the stepwise position). It can be shown (see Refs. [2, 6]) that PDFs of a 1D-PBRW are solutions of the biased telegrapher equation

$$\frac{\partial^2 \rho_X}{\partial t^2} + (\lambda_l + \lambda_r) \frac{\partial \rho_X}{\partial t} + v (\lambda_l - \lambda_r) \frac{\partial \rho_X}{\partial x} = v^2 \frac{\partial^2 \rho_X}{\partial x^2} \tag{A14}$$

where parameters λ_l^{-1} and λ_r^{-1} are the average walk times in the leftward and rightward direction (between alternations), and v is the instantaneous speed (*c.f.* rules 2 and 4). Ensemble averaged displacement (EAD), $\langle X(t) \rangle = \int_{-\infty}^{\infty} x \rho_X(x, t) dx$, can be calculated in two steps. Step one, multiply both sides of Eq. A14 by x. Step two, integrate by parts over the entire x-axis. Assuming the position distribution $\rho_X(x, t)$ and its first spatial derivative $\frac{\partial \rho_X}{\partial x}$ tend to zero as $x \to \pm\infty$, integration by parts gives the ordinary differential equation

$$\frac{d^2 \langle X(t) \rangle}{dt^2} + \lambda_+ \frac{d \langle X(t) \rangle}{dt} = v \lambda_- \tag{A15}$$

where parameters $\lambda_\pm \equiv \lambda_l \pm \lambda_r$ are introduced to simplify the equations. The frequency λ_- defines the degree of directional bias: leftward when $\lambda_- < 0$ and rightward when $\lambda_- > 0$. The frequency λ_+ is inversely related to the average step duration, $t_p = \frac{1}{2} \left(\lambda_l^{-1} + \lambda_r^{-1} \right)$, known as persistence time [2].

Solving Eq. A15 with initial conditions $\langle X(t) \rangle|_{t=0} = 0$ and $\frac{d \langle X(t) \rangle}{dt}|_{t=0} = 0$ gives the EAD

$$\langle X(t) \rangle = v \frac{\lambda_-}{\lambda_+} \left[t - \frac{1}{\lambda_+} \left(1 - e^{-\lambda_+ t} \right) \right]. \tag{A16}$$

Note that when there is no bias (*i.e.* $\lambda_- = 0$), EAD is identically equal to 0.

The EASD, $\langle X^2(t) \rangle = \int_{-\infty}^{\infty} x^2 \rho_X(x, t) dx$, is calculated analogously to the EAD. Multiplying both sides of the biased telegrapher equation (Eq. A14) by x^2 and integrating gives an ordinary differential equation

containing $\langle X(t)\rangle$ and $\langle X^2(t)\rangle$ terms. Substitution of $\langle X(t)\rangle$ using Eq. A16 gives the ordinary differential equation

$$\frac{d^2\left\langle X^2(t)\right\rangle}{dt^2}+\lambda_+\frac{d\left\langle X^2(t)\right\rangle}{dt}=2v^2\left\{1+\frac{\lambda_-^2}{\lambda_+}\left[t-\frac{1}{\lambda_+}\left(1-e^{-\lambda_+ t}\right)\right]\right\}. \tag{A17}$$

Solving for $\langle X^2(t)\rangle$ using the initial conditions $\langle X^2(t)\rangle|_{t=0}=0$ and $\frac{d\langle X^2(t)\rangle}{dt}|_{t=0}=0$ gives the EASD

$$\left\langle X^2(t')\right\rangle=\frac{v^2}{\lambda_+^2}\left[2\left(3c^2-1\right)\left(1-e^{-t'}\right)+2\left(1-c^2\left(2+e^{-t'}\right)\right)t'+c^2t'^2\right] \tag{A18}$$

where $t'\equiv\lambda_+ t$ is nondimensionalized time and $c=\frac{\lambda_-}{\lambda_+}$ is the tangent-bias correlation as described in the previous section (*c.f.* rule 3). When there is bias ($c^2>0$), $\left\langle X^2(t)\right\rangle\to c^2v^2t^2$ in the large time scale limit ($t'\to\infty$), corresponding to directional motion with an RMS speed asymptote

$$v_{\mathrm{rms}}(\infty)=|c|v \tag{A19}$$

just like in the BRW model (model 2). At maximal bias, $c^2=1$, the walker travels in a straight line with speed v. With no bias, $c^2=0$, the t'^2 term vanishes and the t' term indicative of diffusive motion dominates at large time scales ($t'\to\infty$). In this special case, EASD simplifies to the persistent random walk (PRW) equation commonly used to model the movement of chemokinetic cells [2, 7, 8]

$$\left\langle X^2(t)\right\rangle=2v^2t_p\left[t-t_p\left(1-e^{-t/t_p}\right)\right] \tag{A20}$$

where $t_p\equiv\lambda_+^{-1}=\frac{1}{2}\langle T^{(l)}\rangle=\frac{1}{2}\langle T^{(r)}\rangle$ is the so-called persistence time, half the average time the walker moves in one direction before switching to the other.

Differentiating Eq. A18 gives the log-log EASD slope

$$\beta_{\mathrm{PBRW}}(t';\,c)=\frac{(2-4c^2)t'+2c^2t'^2+(4c^2-2)t'e^{-t'}+2c^2t'^2e^{-t'}}{(6c^2-2)+(2-4c^2)t'+c^2t'^2+(2-6c^2)e^{-t'}-2c^2t'e^{-t'}}. \tag{A21}$$

These curves are plotted for multiple values of tangent-bias correlation c in Fig. 2 A (solid curves). Of particular interest is the form of a directionality time that would be calculated from the log-log EASD slope in Eq. A21. Persistence over short time scales causes a $\beta_{\mathrm{PBRW}}(0)=2$ and instead of a monotonic increase of $\beta_{\mathrm{PBRW}}(t)$ from 1 to 2 as with $\beta_{\mathrm{DD}}(t)$ and $\beta_{\mathrm{BRW}}(t)$ (Eqs. A2 and A8, respectively), β_{PBRW} dips towards a value of 1 before eventually asymptoting back towards 2 as $t\to\infty$. At sufficiently large time

scales, the constant terms and terms with decaying exponentials in Eq. A21 becomes negligible and

$$\beta_{\mathrm{PBRW}}(t' > t'_{\mathrm{BRW}}) \simeq \beta_{\mathrm{BRW}}(t';t'_0) = \frac{1+\frac{2t'}{t'_0}}{1+\frac{t'}{t'_0}} \tag{A22}$$

where $t'_0 = \frac{2(1-2c^2)}{c^2}$ (Fig. 2 A, dashed curves). This is known as the β_{BRW}-model. The convergence time scale above which the $\beta_{\mathrm{PBRW}}(t)$ is approximated by the β_{BRW}-model in Eq. A22, denoted t'_{BRW}, is defined as the time above which $\left|\frac{\beta_{\mathrm{PBRW}}(t')-\beta_{\mathrm{BRW}}(t';t'_0)}{\beta_{\mathrm{BRW}}(t';t'_0)}\right| < 5\%$ (Fig. 2 B, solid black curve). For the purpose of fitting the β_{BRW}-model to PBRW data, choosing to fit data at times above the convergence time scale is important. Fitting at times above $10\lambda_+^{-1}$ will produce good fits because $10\lambda_+^{-1} > t_{\mathrm{BRW}}$ for all but the most unbiased motion, in which case directionality time has little meaning regardless.

Notably, at time scales $t' > t'_{\mathrm{BRW}}$, β_{PBRW} has the same functional form as the β_{DD} and β_{BRW} (Eqs. A2 and A8, respectively). Analogous to both DD and the 2D-SBRW, defining directionality time as the time when $\beta_{\mathrm{BRW}} = \frac{3}{2}$ gives $t'_d = t'_0 = \frac{2(1-2c^2)}{c^2}$. In the interpretation that directionality time is a proxy for the observation time necessary to distinguish directionality from randomness, we redefine directionality time to vanish in the $\frac{1}{2} < c^2 < 1$ domain, signifying directional motion at all time scales:

$$t_d = \begin{cases} \frac{1}{\lambda_+}\frac{2(1-2c^2)}{c^2}, & 0 < c^2 < \frac{1}{2} \\ 0 & \frac{1}{2} < c^2 < 1 \end{cases} \tag{A23}$$

(Fig. 2 C). Importantly, directionality time depends only on the reorientation time scale, λ_+^{-1}, and reorientation bias, c^2. Directionality time does not depend on speed v.

Model 3A: 1-D Persistent Biased Random Walk with measurement error (PBRWwE)

The 1D-PBRW (model 3) can be modified to account for measurement errors. Assuming positions along the PBRW are sampled from Normal distribution with standard deviation σ_m (see Fig. 3 A for diagram is 2-D), the change to EASD is: $\langle X^2(t)\rangle \to \langle X^2(t)\rangle + 2\sigma_m^2$, where $\langle X^2(t)\rangle$ is given in Eq. A18. The log-log

EASD slope becomes

$$\beta_{\mathrm{PBRWwE}}(t';\,c) = \frac{(2-4c^2)t' + 2c^2t'^2 + (4c^2-2)t'e^{-t'} + 2c^2t'^2e^{-t'}}{(6c^2-2) + (2-4c^2)t' + c^2t'^2 + (2-6c^2)e^{-t'} - 2c^2t'e^{-t'} + \underbrace{\frac{2\sigma_m^2\lambda_+^2}{v^2}}_{\equiv\epsilon^2}} \tag{A24}$$

where the term added with respect to Eq. A21 is marked by an underbrace and defined as ϵ^2 for brevity. As with the 1-D PBRW without measurement error, the 1-D PBRWwE also converges to $\beta_{\mathrm{BRW}}(t)$. The convergence times above which the difference between β_{PBRWwE} and β_{BRW} is less than 5% are shown in S2A Fig. (solid curves) for different values of the constant ϵ. These convergence times increase with ϵ^2 (*i.e.* increasing centroid measurement error and/or decreasing instantaneous speed). When ϵ^2 is sufficiently large, the convergence time is estimated by comparing ϵ^2 to $c^2t'^2$ (see the denominator of Eq. A24), giving $t_{\sigma_m} \approx \frac{4.5\sqrt{2}\sigma_m}{v_{\mathrm{rms}}(\infty)} = \frac{4.5\,\epsilon}{\lambda_+ c}$ (S2A Fig., dashed curves), where $v_{\mathrm{rms}}(\infty) = |c|v$ as derived in Eq. A19. The factor of 4.5 was chosen so the convergence time scale corresponds to the 5% difference threshold. A larger factor can be chosen to lower the percent difference threshold. This convergence time scale can be used to set a minimum fit time (*c.f.* τ_{min} in Fig. 3 D). Fitting the β_{BRW}-model at times above the minimum fit time significantly decouples directionality time measurements from measurement error. Note that t_{σ_m} is sampling interval independent.

As the dimensionality of the random walk increases, so does the effect of measurement error. Specifically, $\langle X^2(t)\rangle \to \langle X^2(t)\rangle + 2d\sigma_m^2$ where d is the number of dimensions. Also taking into the account the convergence time t_{BRW} due to persistence over short time scales, the generalized convergence time scale, known as the minimum fit time t_{min}, is the greater of:

$$t_{\mathrm{min}} = \max\left\{\begin{matrix} t_{\sigma_m} \approx \frac{4.5\sqrt{2d}\sigma_m}{v_{\mathrm{rms}}(\infty)} \\ t_{\mathrm{BRW}} \approx 10\frac{1}{\lambda_+} \approx 10t_p \end{matrix}\right\} \tag{A25}$$

both of which are readily measured and sampling interval independent. Fitting experimental data to the β_{BRW}-model at times above t_{min} decouples measurements of directionality time, t_d, from the effects of persistence and measurement error.

Above we have shown that directionality time can be derived as long as t_d is not too small compared to t_{σ_m} such that information about directionality is hidden beneath large measurement errors. Fitting at times above t_{min} ensures that the directionality time measurement decouples from measurement error and

persistence time. One final case to explicitly consider is that when directionality time is large compared to duration of data collection. One potential concern is the possibility that fitting the β_{BRW}-model renders a measure of the transition from pseudo random kinematics caused my measurement noise, to persistent but not necessarily directed motion. This case corresponds to a log-log MSD slope estimated by taking Eq. A24 and setting $c = 0$ (*i.e.* t_d much larger than the time scale of data collection). The corresponding asymptotic form of log-log MSD slope is

$$\beta_{\mathrm{PBRWwE}}(t'; \, c = 0) \simeq \frac{t'}{\frac{1}{2}\epsilon^2 + t'} \tag{A26}$$

which is a $\beta = 0$ to $\beta = 1$ transition that is not captured by the β_{BRW}-model. When Eq. A26 is fit to the β_{BRW}-model at times above t_{min}, the directionality time measurement approaches infinite, therefore proving that the β_{BRW}-model does not erroneously couple to a time scale other than that associated with directional bias.

Appendix B: Deviations Caused By Variances and Nonergodicity

In Appendix A, we showed that biased random walks could be described by the β_{BRW}-model

$$\beta_{\mathrm{BRW}}(t;\ t_d) = \frac{1+\frac{2t}{t_d}}{1+\frac{t}{t_d}} \tag{B1}$$

which can be used to measure directionality time, t_d. This analytical fit model implicitly accounts for the variance of position, $\mathbf{R}$, caused by variance in the step time distribution, $\rho_T(t)$, and the orientation distribution, $\rho_\Theta(\theta)$ (*c.f.* Appendix A, rules 2 and 3). However, it does not account for other sources of variance associated with measurements of real data, or variance of parameters such as speed, v, which can take on a different value for each random walker in an ensemble. We will refer to these three categories of variances as: implicit variance, measurement error, and parametric variance, respectively. The implicit variance is given by, $\sigma_R^2(t) = \langle R^2(t)\rangle - \langle R(t)\rangle^2$. The variance attributed to measurement error is $2d\sigma_m^2$, where d is the number of dimensions. Finally, parametric variance is denoted $\sigma_p^2(t)$. Adding the variances in quadrature and using the 1D-PBRW model to calculate implicit variance, $\langle X^2(t)\rangle - \langle X(t)\rangle^2$, from Eqs. A16 and A18, the composite variance of position X is

$$\sigma_{X,real}^2(t) = \underbrace{\frac{v^2}{\lambda_+^2}(5c^2-2) + \frac{2v^2}{\lambda_+}(1-c^2)t + \mathrm{O}\left(e^{-\lambda_+ t}\right) + \mathrm{O}\left(te^{-\lambda_+ t}\right)}_{\text{implicit variance, } \sigma_X^2} + \underbrace{2\sigma_m^2}_{\text{measurement variance}} + \underbrace{c^2(\delta v)^2 t^2}_{\text{parametric variance, } \sigma_p^2} \tag{B2}$$

where $\mathrm{O}(f(t))$ denotes a term of order $f(t)$. Note here that $d = 1$. For demonstrative purposes, only one type of parametric variance is written in Eq. B2, the speed variance (at large time scales). There can be other parametric variance terms but in general, $\sigma_p^2(t) \sim t^2$. Therefore, at large time scales, parametric variance is the dominant cause of position variance.

The EASDs derived analytically in Appendix A assumed zero measurement error and parameter variance. When measurement error and parameter variance are nonzero as is the case with experimental

measurements, EASD is increased with respect to our analytical models

$$\langle R^2(t)\rangle = \langle R^2(t)\rangle_{\text{BRW}} + \sigma^2(t) \tag{B3}$$

where $\sigma^2(t) \equiv 2d\sigma_m^2 + \sigma_p^2(t)$ incorporates all measurement and parameter uncertainties, and it has been assumed that each of these uncertainties is random and does not change $\langle R(t)\rangle$.

Ensemble averaged data is often noisy. As more migration trajectories are tracked, the standard errors of ensemble averaged statistics decrease. However, ensemble averaged statistics such as EASD are often still too noisy to fit with many experimental designs. Statistical noise can be reduced by first time averaging the data before calculating the ensemble average. The time averaged squared displacement (TASD), for example, is given by

$$\overline{R_i^2}(\tau) = \frac{1}{(t_{\text{dur},i} - \tau)} \int_0^{(t_{\text{dur},i}-\tau)} |\mathbf{R}_i(t+\tau) - \mathbf{R}_i(t)|^2 \, dt \tag{B4}$$

where i is an integer path index (*i.e.* path 1, 2, 3,...) and $t_{\text{dur},i}$ is the total duration of the ith path. The difference between TASD and EASD is described by

$$\overline{R_i^2}(\tau) = \xi_i(\tau)\langle R^2(t=\tau)\rangle \tag{B5}$$

where the factor ξ_i is known as ergodicity. Mean squared displacement (MSD) is calculated by ensemble averaging TASD (averaging over all trajectories, i)

$$\langle\overline{R^2}(\tau)\rangle = \langle\xi_i(\tau)\rangle\langle R^2(t=\tau)\rangle \tag{B6}$$

For β-curves measured from MSD instead of EASD is

$$\beta(\tau) = \frac{d\,\log\left[\langle\overline{R^2}(\tau)\rangle\right]}{d\,\log[\tau]}. \tag{B7}$$

Rewriting $\langle\overline{R^2}(\tau)\rangle$ in terms ξ, $\langle R^2\rangle_{\text{BRW}}$, and σ^2 using Eqs. B6 and B3 gives

$$\beta(\tau) = \frac{d\,\log\left[\langle\xi(\tau)\rangle\langle R^2(\tau)\rangle_{\text{BRW}}\left(1 + \frac{\sigma^2(\tau)}{\langle R^2(\tau)\rangle_{\text{BRW}}}\right)\right]}{d\,\log[\tau]}. \tag{B8}$$

The log-log MSD slope can thus be expanded into its final form

$$\beta(\tau) = \beta_{\mathrm{BRW}}(\tau) + \beta_\sigma(\tau) + \beta_\xi(\tau) \tag{B9}$$

where $\beta_{\mathrm{BRW}}(\tau)$ is given by Eq. B1, $\beta_\sigma(\tau)$ is given by

$$\beta_\sigma(\tau) = \frac{d\,\log\left[1 + \frac{\sigma^2(\tau)}{\langle R^2(\tau)\rangle_{\mathrm{BRW}}}\right]}{d\,\log[\tau]} \tag{B10}$$

and $\beta_\xi(\tau)$ is given by

$$\beta_\xi(\tau) = \frac{d\,\log\left[\langle \xi(\tau)\rangle\right]}{d\,\log[\tau]}. \tag{B11}$$

The variance correction $\beta_\sigma(\tau)$ becomes negligible as time increases unless $\sigma^2(\tau)$ is of order τ^2 or greater such that $\sigma^2(\tau) \to \infty$ faster than $\langle R^2(\tau)\rangle_{\mathrm{BRW}} \to \infty$. At large time scales, the position variance is dominated by parametric variance and not intrinsic variance (*c.f.* Eq. B2). Therefore, the standard deviations of the mean distance traveled can be used to calculate $\sigma^2(\tau)$ in the variance correction term, $\beta_\sigma(t)$. At large time scales, the variance correction term can be completely determined from the experimental data by using Eq. B3 to substitute $\langle R^2(\tau)\rangle_{\mathrm{BRW}}$ for $\langle R^2(\tau)\rangle - \sigma^2(\tau)$ into Eq. B10, giving

$$\beta(\tau) - \frac{d\,\log\left[\frac{\langle \overline{R^2}(\tau)\rangle}{\langle \overline{R^2}(\tau)\rangle - \overline{\sigma^2}(\tau)}\right]}{d\,\log[\tau]} - \frac{d\,\log\left[\langle \xi(\tau)\rangle\right]}{d\,\log[\tau]} = \beta_{\mathrm{BRW}}(\tau). \tag{B12}$$

Here, all terms that can be measured experimentally have been moved to the left of the equality, while the fit model has been moved to the right. Eq. B12 is only valid at sufficiently large time intervals when the effects of persistence on $\beta(\tau)$ are negligible (*i.e.* $\beta_{\mathrm{PBRW}}(\tau) \approx \beta_{\mathrm{BRW}}(\tau)$, *c.f.* Eqs. A21 and A8), and when the effect of implicit variance is small relative to the combined measurement and parametric variances (*i.e.* $\left[\langle \overline{r^2}(\tau)\rangle - \langle \overline{r}(\tau)\rangle^2\right] \ll \left[\sigma_m^2 + \sigma_p^2(\tau)\right]$, *c.f.* Eq. B2). The minimum fitting time interval $\tau_{\min}$ defined in Eq. A25 can be used to satisfy these conditions.

As a final note, calculating the ergodicity correction term $\beta_\xi(\tau)$ from experimental data is not always helpful because its uncertainty is often relatively large and ergodicity cannot be time averaged to reduce statistical noise. Therefore, the correction term $\beta_\xi(\tau)$ should only be calculated if the system is believed to be nonergodic (*i.e.* when the parameters that describe an experimentally measured migration change significantly over the duration of that migration). If the data is nonergodic and $\beta_\xi(\tau)$ is unacceptably

noisy when calculated from experimental data, sometimes truncating the migration paths to equalize (and/or shorten) their durations will make the data nearly ergodic. This was the procedure used in order to ignore the ergodicity correction term ($\beta_\xi(\tau) = 0$) in the chemotactic neutrophil example from the main text (Fig. 4).

If the data is still nonergodic after truncation, a quick and dirty first approach of choosing $\beta_\xi = a$, where a is a constant such that $\lim_{\tau\to\infty} [\beta(\tau) - a] = 2$, may reasonably capture the nonergodic correction. A more precise calculation of $\beta_\xi(\tau)$ can be achieved by simulating biased random walks as follows. First, make an initial measurement of the persistence time and directionality time from the truncated migration paths. Based on these initial measurements, parameters t_p and $\kappa = \kappa(c^2)$ can be chosen for a corresponding 2D-PBRW simulation that will recapitulate the experimental data. Second, calculate the ensemble averaged instantaneous speed (EAIS) of the truncated migration paths over their entire duration $0 \le t \le t_{\mathrm{dur}}$. When the data is nonergodic, there will typically be an overall acceleration or deceleration associated with the EAIS. Third, run 2D-PBRW simulations with instantaneous speeds, $V(t)$, randomly selected from the experimentally measured EAIS distribution each time a reorientation event occurs. Simulate enough trajectories so that a smooth ensemble averaged ergodicity curve as a function of time interval, $\xi(\tau)$, can be calculated. Finally, calculate ergodicity correction term $\beta_\xi(\tau)$ using Eq. B11.

References

1. Sze SM, Lee MK. Semiconductor Devices: Physics and Technology. 3rd ed. Wiley; 2012.

2. Othmer HG, Dunbar SR, Alt W. Models of dispersal in biological systems. J Math Biol. 1988;26:263–298.

3. Evans M, Hastings N, Peacock B. Statistical Distributions. 3rd ed. Wiley; 2000.

4. Marsh LM, Jones RE. The form and consequences of random walk movement models. J Theor Biol. 1988;133:113–131.

5. Codling EA, Plank MJ, Benhamou S. Random walk models in biology. J R Soc Interface. 2008;5:813–834.

6. Goldstein S. On diffusion by discontinuous movements, and on the telegraph equation. J Mech Appl Math. 1951;6:129–156.

7. Parkhurst MR, Saltzman WM. Quantification of human neutrophil motility in three-dimensional collagen gels. Effect of collagen concentration. Biophys J. 1992;61:306–315.

8. Reinhart-King CA, Dembo M, Hammer DA. Cell-Cell mechanical communication through compliant substrates. Biophys J. 2008;95:6044–6051.
